# Oxidation and Reduction of Hydrazones—Risk Factors Related to the Manufacture and Stability of the Drugs

**DOI:** 10.3390/ijms26094295

**Published:** 2025-05-01

**Authors:** Anna B. Witkowska, Krzysztof Stolarczyk, Massimo Fusaro, Andrzej Leś, Joanna Giebułtowicz, Elżbieta U. Stolarczyk

**Affiliations:** 1 Spectrometric Methods Department, National Medicines Institute, Chełmska 30/34, 00-725 Warsaw, Poland; a.witkowska@nil.gov.pl; 2 Department of Drug Chemistry, Pharmaceutical and Biomedical Analysis, Medical University of Warsaw, 61 Żwirki i Wigury, 02-091 Warsaw, Poland; j.giebultowicz@wum.edu.pl; 3 Faculty of Chemistry, University of Warsaw, 1 Pasteura Street, 02-093 Warsaw, Poland; kstolar@chem.uw.edu.pl (K.S.); maxitp@gmail.com (M.F.); ales@chem.uw.edu.pl (A.L.)

**Keywords:** oxidation, reduction, stability, electrochemistry, DFT, in silico methods

## Abstract

This study aimed to evaluate the use of electrochemistry to generate the oxidation and reduction products of active pharmaceutical ingredients (APIs) with a hydrazone group, including dantrolene, nitrofurantoin, furazidine, and nitrofural. In the first step, cyclic voltammetry was employed to assess the electroactivity of these compounds. In the second step, the transformation products of selected APIs following electrochemical oxidation and reduction were analyzed using the ROXY EC System equipped with a µ-PrepCell™ 2.0, coupled with a high-resolution Q-TOF mass spectrometer. The identification of transformation products was based on accurate mass, isotopic distribution, and fragmentation pattern. Seventeen API impurities were identified in this study, contributing to insights into drug stability and potential risks associated with their manufacture. Experimental findings were supported by the quantum mechanical DFT calculations of the molecular energies. In addition, using commercially available in silico software, the predicted metabolic products were compared with those obtained by experimental methods. The electrochemical approach proved useful as a test for determining the stability of compounds, the detection of new impurities and structure determination using high-resolution mass spectrometry.

## 1. Introduction

Knowledge of drug degradation and metabolism pathways is essential to ensure the safety and efficacy of drug therapy for patients. The global crisis related to the contamination of medical products with carcinogenic *N*-nitrosamines and nitrosamine drug substance-related impurities (NDSRIs), ongoing since 2018, has highlighted the urgent need to develop new methods to predict potentially toxic drug impurities and to control them using appropriate techniques [1]. Bringing a drug product to the market requires extensive stability studies to confirm its quality, safety, efficacy, and stated shelf life. It is of paramount importance to predict as early as possible what may occur during the lifetime of a pharmaceutical product, as this information can significantly shape product development strategies [2]. Electrochemistry has been effectively utilized to replicate the drug metabolism processes typically mediated by cytochrome P450 enzymes. This is accomplished through a series of reactions simulated using electrochemical methods [3,4,5]. Examples of metabolic reactions mimicked by electrochemical approaches include O-dealkylation [6], dehydrogenation [7], benzyl hydroxylation [8], N,S-oxidation [9], N-dealkylation [10], aromatic ring hydroxylation [11], allylic and aliphatic hydroxylation, and hydroxylation [12]. Electrochemistry (EC) is a rapidly evolving analytical tool in the field of predicting drug oxidation/reduction and degradation products, thereby aiding in the assessment of drug stability. Predicting drug degradation by EC-MS can help identify new, potentially concerning impurities such as nitroso-compounds [13]. Over the past four decades, the applications of EC-MS have expanded significantly.

Recent advancements in combining electrochemistry with mass spectrometry include studies on the following:

- drug metabolism [4,12,14];
- characterization of the reducing potencies of antioxidants [15];
- synthesis of pharmaceutical oxidation products [16];
- peptide, protein, and nucleotide research [17,18,19];
- hydrogen/deuterium exchange [20];
- prediction of transformation products in the environment [21];
- investigation of the oxidative stability of drugs [22,23].

The processes that occur in living organisms are fundamentally redox reactions involving oxidation and reduction. In EC-MS, the substances can be oxidized or reduced on the surface of a working electrode depending on the potential applied voltage, making this technique a promising tool for mimicking oxidative Phase I and even conjugative Phase II metabolism. EC studies can be conducted in two modes, which are online and offline. The online mode enables the detection of both stable and short-lived species thanks to the direct connection of the electrochemical cell to the mass spectrometer, while in offline mode, oxidation/reduction products and intermediates are collected and analyzed later [3] (Figure S1).

Various types of electrochemical reactors are employed in EC-MS research, with the most common being amperometric thin-layer cells and wall-jet electrochemical cells. Coulometric flow-through cells measure changes in generated potential, whereas amperometric cells measure the generated current [13]. Increasing attention is also being given to microfluidics and microchips [24,25]. Each electrochemical reactor is equipped with the following three electrodes: a reference electrode, an auxiliary electrode (AUX), and a working electrode (glassy carbon, titanium, platinum, gold or boron-doped diamond). The boron-doped diamond (BDD) electrode is particularly valued for its exceptional stability in aggressive environments and chemical inertness [26].

Over time, a wide variety of systems incorporating EC have been employed in research, the most popular currently being the coupling of electrochemistry with mass spectrometry (mainly high-resolution-HR), capillary electrophoresis with MS, and liquid chromatography with MS [17]. High-resolution mass spectrometry is the optimal choice for determining the exact masses of compounds generated in the EC flow cell and fragmentation patterns, facilitating the prediction of oxidation and reduction product structures. The electrospray ionization (ESI) source is the most commonly used in EC-HRMS coupling, offering high sensitivity and reliability at low flow rates [27,28,29,30,31].

As previously mentioned, EC-MS coupling has been applied in numerous contexts, yet no studies to date have explored the stability of drugs with hydrazone groups. The oxidation and reduction of hydrazones, hydrazides, and hydrazine-type compounds can lead to the formation of hazardous impurities [32,33,34], underscoring the urgent need to monitor the oxidation and reduction products of drugs containing a hydrazone group as part of risk analysis for their manufacture and stability. Electrochemistry has already been established as a tool for oxidative and reductive stress testing, proving to be a faster alternative that does not require strong oxidants [2]. Understanding how oxidative stress affects a drug enables the determination of optimal storage conditions (e.g., temperature, humidity, light exposure) to maximize shelf life and prevent premature degradation. Oxidative degradation can produce byproducts that may be toxic or cause unwanted side effects. Understanding these pathways helps identify and mitigate potential risks to patients.

**Figure 1:**
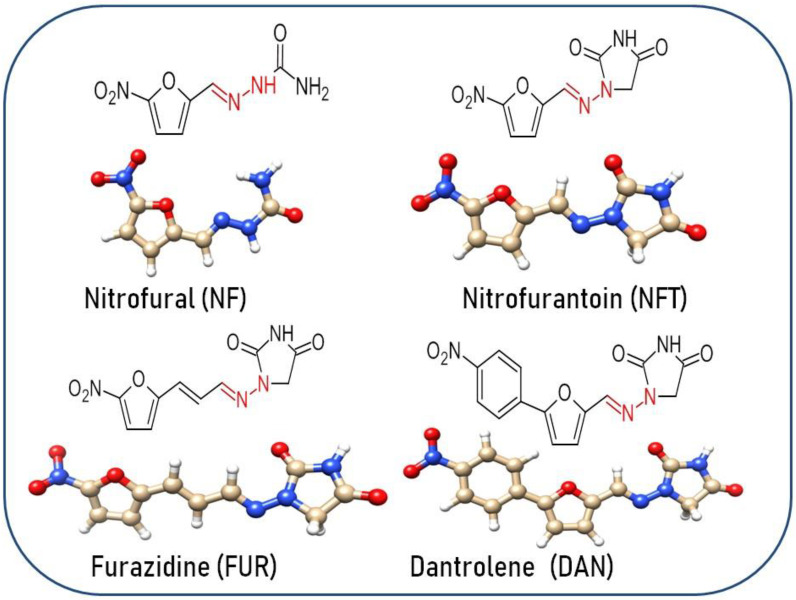
Structure of selected APIs for study.

Our research is focused on impurities that may form following the oxidation/reduction of active pharmaceutical ingredients (APIs) with a hydrazone group, such as dantrolene, nitrofurantoin, furazidine, and nitrofural (nitrofurazone) (Figure 1).

The process of mineralization of nitrofural in water in the presence of chloride ions by electrochemical treatment has been previously studied by Kuznetsov et al. [35]. In this study, complete electrochemical destruction of nitrofural was achieved on BDD anodes after 30 min of treatment, and during that process, four oxidation products were identified, which were inter alia dichloro-derivatives of nitrofural. In the literature, one oxidation metabolite of nitrofural is known, which is 4-hydroxynitrofurazone, while the main product of its metabolism is semicarbazide, formed after reduction by azoreductase, and hydroxylamine is also produced [36,37,38]. For nitrofurantoin, one impurity is known following 4-hydroxylation, and reduction by nitroreductase may occur during metabolism, with the primary metabolite being 1-aminohydantoin [39,40]. For furazidine and dantrolene, oxidation and reduction products have not yet been investigated using EC-MS. Experimental data on the reduction and oxidation products of furazidine are not available in the literature. Dantrolene is metabolized to 5-hydroxydantrolene and may also undergo the reduction of its phenyl nitro group to aminodantrolene [41,42].

Due to the limited knowledge about the degradation products of APIs containing a hydrazone group, we aimed to apply a comprehensive approach using EC-MS-QTOF, DFT, and in silico metabolite simulations to identify novel API impurities. The EC-MS-QTOF combination employed provided rapid, reliable, and detailed structural information on drug metabolites and impurities. DFT calculations and in silico metabolite simulations further confirmed and complemented the electrochemical findings.

## 2. Results and Discussion

Electrochemistry has been applied to the pharmaceutical stability testing of DAN, FUR, NF, and NFT—active pharmaceutical ingredients—as model substances with hydrazone groups, with results compared to in silico predictions. Compounds with nitrogenous fragments in their structure can be a source of dangerous impurities and, therefore, should be studied and controlled. The ICH M7 guideline designates N-nitroso and alkyl-azoxy compounds, alongside aflatoxin-like substances, as a “cohort of concern” due to their potential mutagenic and carcinogenic risks [43].

Initial studies were conducted using electrochemical methods. This study also aimed to investigate whether the coupling of EC-HR/MS is a useful tool to quickly and easily elucidate degradation products and estimate drug stability for drugs with hydrazone group. Additionally, degradation products predicted using commercially available in silico software were compared to those obtained from the electrochemical methods.

### 2.1. Electrochemical Measurements

As a first step, electrochemical tests were performed to investigate whether the tested compounds are electroactive. The study using cyclic voltammetry was performed using a GCE electrode in the presence of furazidine (FUR), dantrolene (DAN), nitrofural (NF), and nitrofurantoin (NFT) in deoxygenated solutions of acetonitrile and ammonium formate.

**Figure 2:**
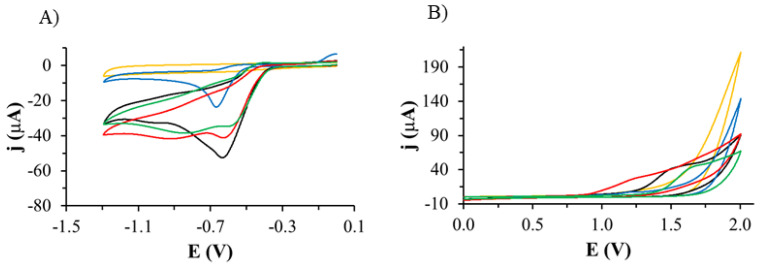
Cyclic voltammetric curves recorded using the GCE electrode in the range of potential: (**A**) from 0 V to −1.3 V and (**B**) from 0 V to 2.0 V in ACN/ammonium formate solution with a pH of 7.4 (yellow curve) containing 1 mM of FUR (black curve), 1 mM of DAN (blue curve), 1 mM of NF (red curve), and 1 mM of NFT (green curve), scan rate of 0.1 V/s.

The first studies were performed in the potential range from 0 V to −1.5 V in the deoxygenated basic electrolyte solution. On the voltammetric curve (Figure 2A) recorded using the GCE electrode in the ACN/ammonium formate solution pH 7.4, no signals were observed in the potential range tested. In the deoxygenated solution of 1 mM FUR, a peak was observed on the voltammetric curve recorded in the solution at a potential of −0.635 V with a current value of −52.5 µA; for 1 mM of DAN, there was a peak at a potential of −0.666 V with a current value of −23.7 µA; for 1 mM of NF, there were peaks at potentials of −0.627 V with a current value of −41.2 µA and −0.925 V with a current value of −41.8 µA; and for 1 mM of NFT, there was a peak at a potential of −0.607 V with a current value of −34.8 µA and a peak at a potential of −0.851 V with a current value of −38.5 µA. All processes were irreversible.

Then, the tests were performed in the potential range from 0 V to 2.0 V in the basic electrolyte solution without the active substance (Figure 2B). No signals were observed on the voltammetric curves recorded in this potential range. In the solution containing 1 mM of FUR, a peak was observed on the voltammetric curve at a potential of 1.551 V with a current value of 41.5 µA; for 1 mM of DAN, there was a peak at a potential of 1.306 V with a current value of 16.6 µA; for 1 mM of NF, there was a peak at a potential of 1.222 V with a current value of 24.5 µA; and for 1 mM of NFT, a peak was observed at a potential of 1.732 V with a current value of 61.6 µA. All processes were irreversible.

All the compounds tested are electrochemically active, as they undergo both oxidation and reduction in the potential ranges tested. This is manifested by the presence of corresponding peaks on the recorded voltammetric curves. This is consistent with the literature, where the presence of signals on the voltammetric curves recorded in aqueous solutions is related to the presence of easily reducible nitro groups in the structure of their molecules [44,45,46,47,48].

The electroreduction mechanisms of these compounds are quite complex and involve several electrochemical steps. Ultimately, this leads to the formation of a hydroxylamine derivative (RNHOH) or an amine (RNH_2_). The electrochemical reduction processes of these compounds depend on both the electrode material and the pH of the solution. As a result of oxidation, intermediate oxidation products such as dimer, trimer, or polymers can be formed. Oxidative polycondensation reactions very often occur with these types of compounds [35].

Cyclic voltammetry cannot identify the structure of oxidation or reduction products; therefore, for the next part of the work, the ROXY system coupled with MS was used.

### 2.2. Electrochemical Measurements ROXY-QTOF

**Figure 3:**
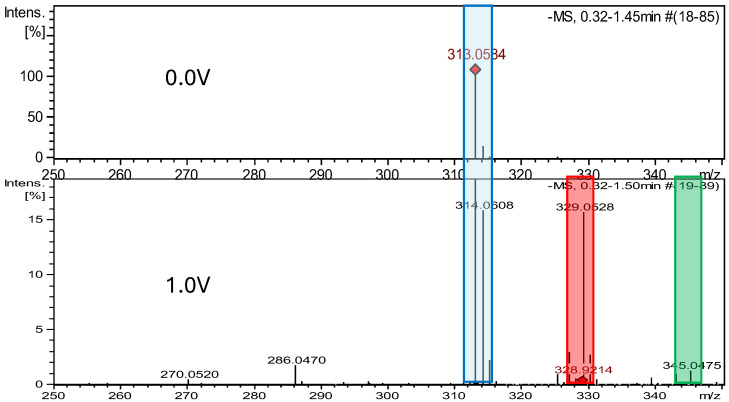
Comparison of ESI–MS spectra of oxidation products of dantrolene at 0.0 V and 1.0 V. Blue—dantrolene, red—DAN-Imp1-Ox, and green—DAN-Imp2-Ox.

**Figure 4:**
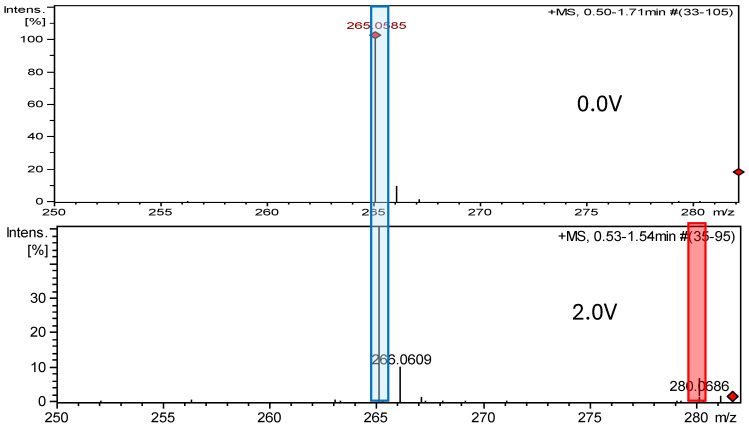
Comparison of ESI–MS spectra of oxidation products of furazidine at 0.0 V and 2.0 V. Blue—furazidine, and red—FUR-Imp1-Ox.

**Figure 5:**
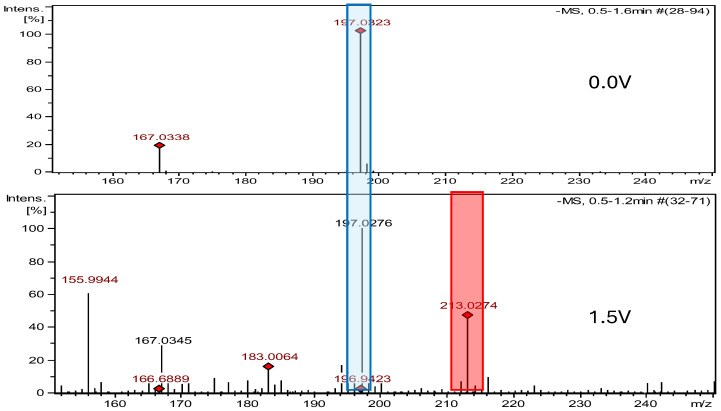
Comparison of ESI–MS spectra of oxidation products of nitrofural at 0.0 V and 1.5 V. Blue—nitrofural, and red—NF-Imp1-Ox.

**Figure 6:**
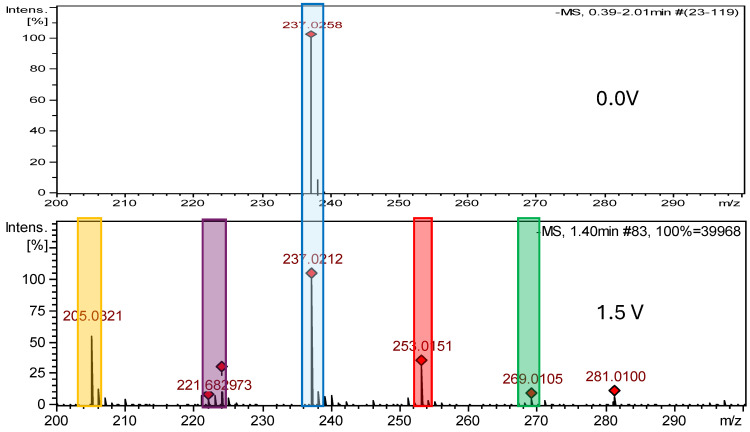
Comparison of ESI–MS spectra of oxidation products of nitrofurantoin at 0.0 V and 1.5 V. Blue—nitrofurantoin, red—NFT-Imp1-OX, purple—NFT-Imp2-OX, green—NFT-Imp3-OX, and yellow—degradation impurity.

**Figure 7:**
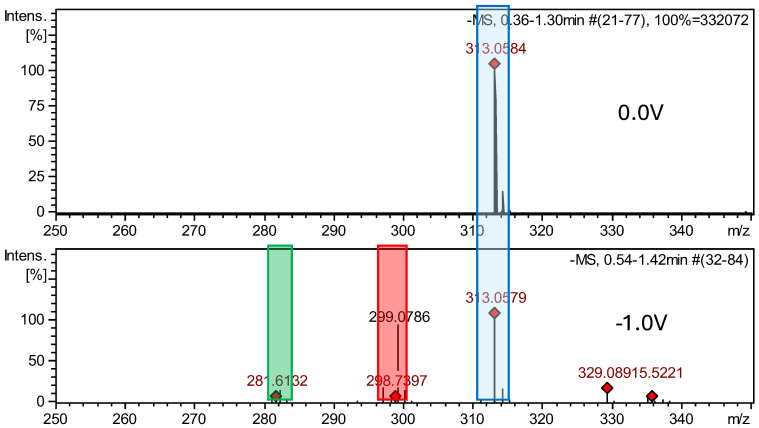
Comparison of ESI–MS spectra of reduction products of dantrolene at 0.0 V and −1.0 V. Blue—dantrolene, red—DAN-Imp1-RED, and green—DAN-Imp2-RED.

**Figure 8:**
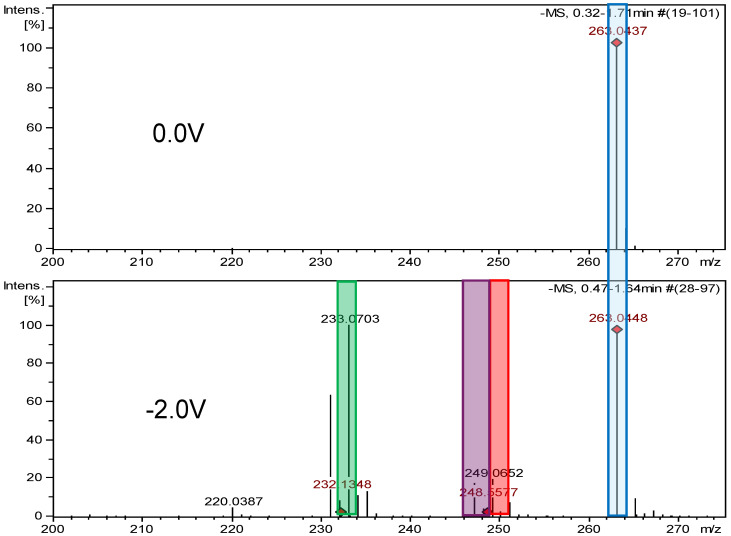
Comparison of ESI–MS spectra of furazidine’s reduction products at 0.0 V and −2.0 V. Blue—furazidine, green—FUR-Imp1-RED, purple—FUR-Imp2-RED, and red—FUR-Imp3-RED.

**Figure 9:**
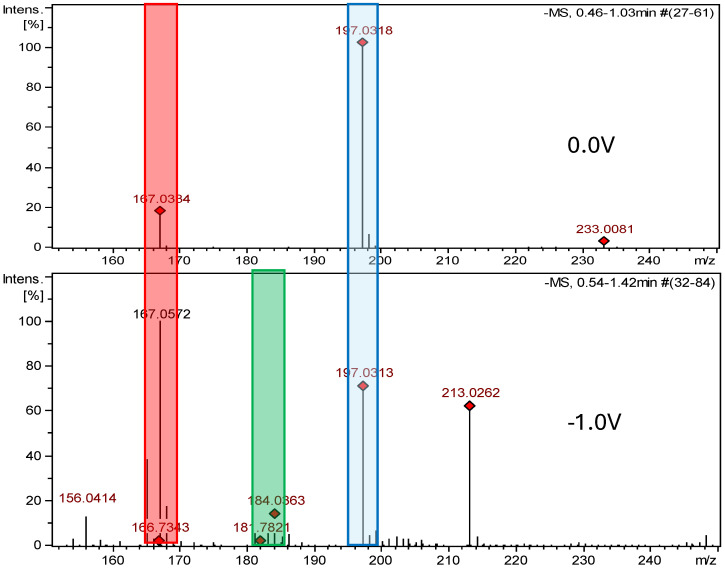
Comparison of ESI–MS spectra of nitrofural’s reduction products at 0.0 V and −1.0 V. Blue—nitrofural, red—NF-Imp1-RED, and green—NF-Imp2-RED.

**Figure 10:**
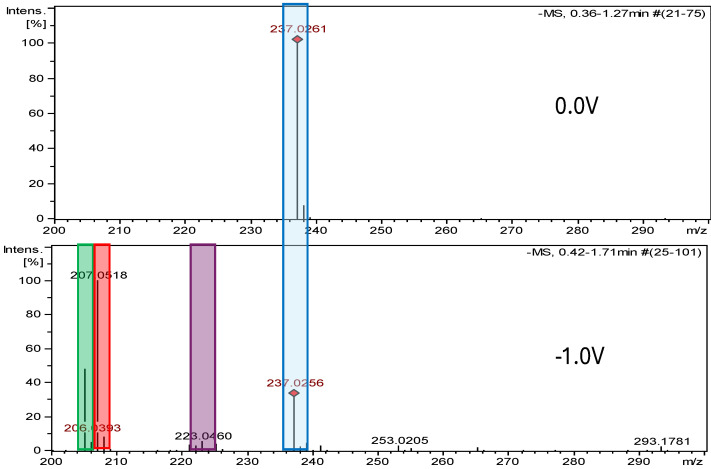
Comparison of ESI–MS spectra of nitrofurantoin’s reduction products at 0.0 V and −1.0 V. Blue—nitrofurantoin, red—NFT-Imp1-RED, green—NFT-Imp2-RED, and purple—NFT-Imp3-RED.

The ROXY electrochemical reactor cell (EC) was used to generate reduction and oxidation API impurities. Measurements were made at different potentials (from 0.0 V to 2.0 V for oxidation, from 0.0 V to −2.0 V for reduction). Selected compounds were subjected to electrochemical degradation, and the differences in resulting products were analyzed. Figure 3, Figure 4, Figure 5 and Figure 6 show ion chromatograms (MS spectra) recorded at oxidation potentials for DAN, FUR, NF, and NFT, respectively. Figure 7, Figure 8, Figure 9 and Figure 10 show ion chromatograms (MS spectra) recorded at reduction potentials for DAN, FUR, NF, and NFT, respectively. A total of seven impurities under oxidation conditions and ten impurities under reduction conditions were obtained and identified. The APIs themselves were used as templates for the interpretation of the unknown structures of products formed during electrochemical degradation. Many product ions were observed in the HR-MS/MS spectra of the pseudomolecular ions of DAN FUR, NF, and NFT (Figures S2–S5). These data were used to propose the structures and fragmentation pathways for the detected impurities. The proposed structures of certain identified impurities are supported by the literature [35,36,37,38,39,40,41,42].

#### 2.2.1. Electrochemical Oxidation

**Figure 11:**
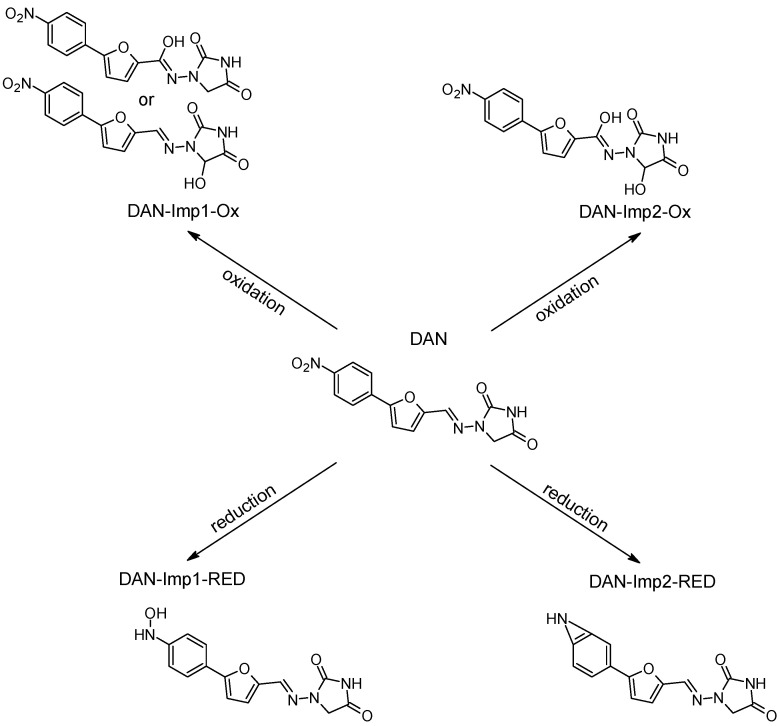
Proposed degradation products of DAN.

**Figure 12:**
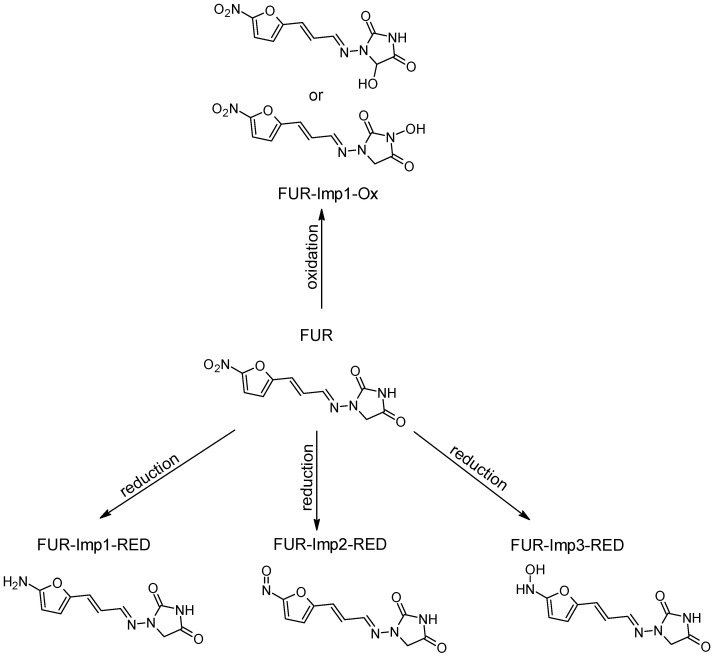
Proposed degradation products of FUR.

**Figure 13:**
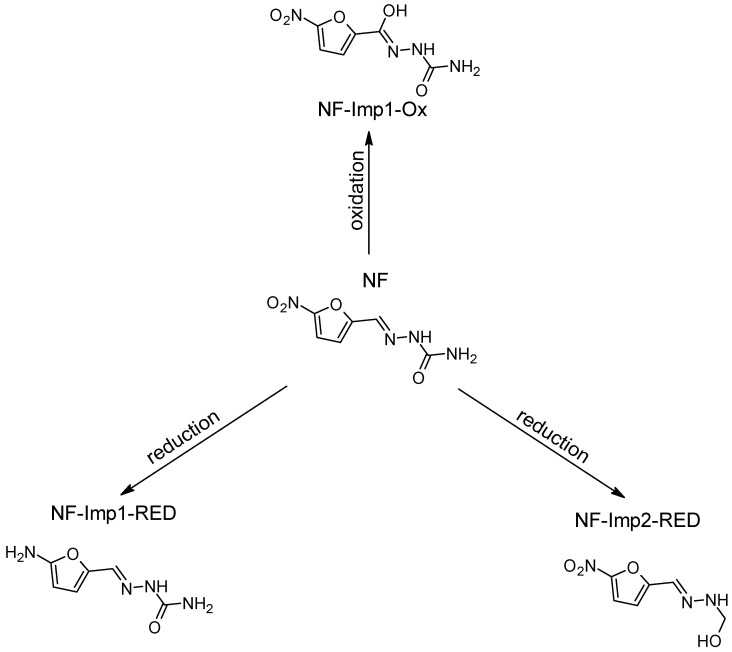
Proposed degradation products of NF.

**Figure 14:**
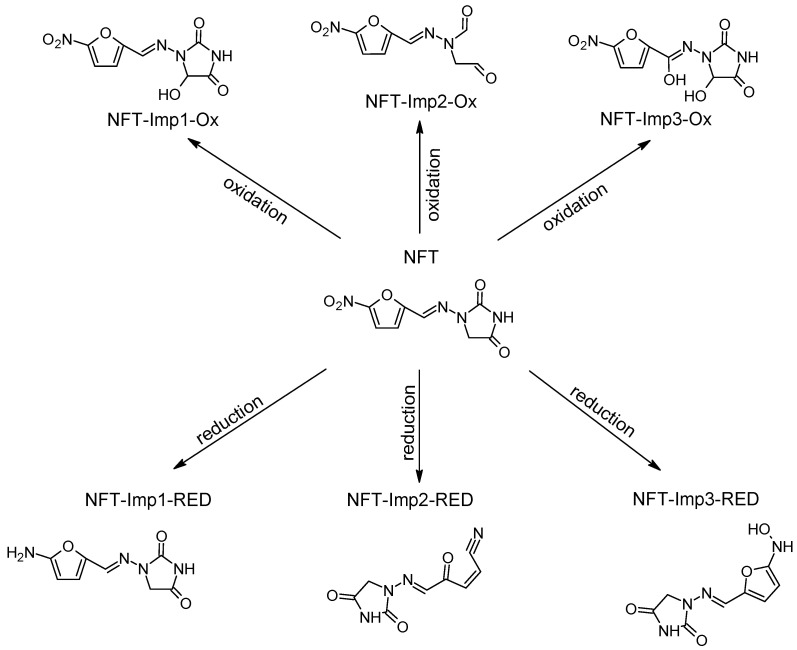
Proposed degradation products of NFT.

The formation of the API electrochemical oxidation products was monitored by ESI-Q-TOF. The results are summarized in Table 1, and the proposed structures are shown in Figure 11, Figure 12, Figure 13 and Figure 14. APIs, without being subjected to oxidation, were analyzed as a control sample.

After electrochemical oxidation, two dantrolene oxidation products not previously described in the literature were detected, which were DAN-Imp1-Ox and DAN-Imp2-Ox. The HRMS analysis of MS spectra showed deprotonated molecular ion peaks at *m*/*z* 329.0528 [M−H]^−^ and *m*/*z* 345.0475 [M−H]^−^, corresponding to the molecular formulas C_14_H_10_N_4_O_6_ and C_14_H_10_N_4_O_7_, respectively. DAN-Imp1-Ox may be identified with two isomeric structures denoted as DAN-Imp1-Ox(A) and DAN-Imp1-Ox(B). With the DFT calculations, it was estimated that there was a small ΔG difference of 5 kcal/mol between the A- and B-isomers, pointing to isomer A as a preferable structure. According to the literature, a dantrolene metabolite has been identified as 5-hydroxydantrolene, formed by the oxidation of the hydantoin ring, which is also confirmed by our in silico studies. However, in our electrochemical studies, based on the fragmentation obtained, oxidation occurs also at the aliphatic residue [35]. It was observed that, under these experimental conditions, an applied voltage of 1.0 V was the optimal potential for generating DAN-Imp1-Ox, and 1.2 V was optimal for DAN-Imp2-Ox. Figures S6 and S7 display the MS2 spectra of dantrolene’s impurities, while Figures S23 and S24 present the structures of characteristic fragments, confirming their identification.

HRMS analysis of MS spectra of the oxidized furazidine solution at 2.0 V showed one new, previously unreported oxidation product, FUR-Imp1-Ox. The MS spectrum showed a protonated molecular ion peak at *m*/*z* 280.0686 [M +H]^+^, corresponding to the molecular formula C_10_H_8_N_4_O_6_. The FUR-Imp1-Ox(A) structure has a lower Gibbs energy and is observed as a predicted metabolite. However, the MS2 spectrum (Figure S8) of this impurity and the structures of the characteristic fragments (Figure S25) confirm the identification of the FUR-Imp1-OX(B) structure.

In the case of nitrofural, oxidation of the sample at 0.7 V led to the formation of one previously unknown oxidative degradation product, which was NF-Imp1-Ox. The HRMS analysis of MS spectra showed a deprotonated molecular [M−H]^−^ ion at *m*/*z* 213.0274, corresponding to the molecular formula C_6_H_5_N_4_O_5_. Figure S9 presents the MS2 spectrum of this impurity, while Figure S26 provides the structures of characteristic fragments, confirming their identification. In nitrofural, electrochemical oxidation occurs at the carbon of the aliphatic chain.

After the electrochemical oxidation reaction of nitrofurantoin, the following three new degradation products were detected: NFT-Imp1-Ox, NFT-Imp2-Ox and NFT-Imp3-Ox. MS spectra showed that, at 800 mV, the first hydroxylation product was formed (oxidation in hydantoin ring) with a molecular ion peak at *m*/*z* 253.0226 [M−H]^−^; at 1.2 V, dioxidation occurs: *m*/*z* 269.0177 [M−H]^−^. The oxidation of nitrofurantoin also proceeded with the opening of the imidazolidine-2,4-dione ring: an ion peak at *m*/*z* 223.0323 [M−H]^−^. Figures S10–S12 display the MS2 spectra of nitrofurantoin’s impurities, while Figures S27–S29 present the structures of characteristic fragments, confirming their identification.

A comparison of the oxidation products obtained by the electrochemical method and the metabolites predicted in silico is summarized in Table 1.

#### 2.2.2. Electrochemical Reduction

The reduction of dantrolene, nitrofurazone, nitrofurantoin, and furazidine may involve different mechanisms and conditions described in the literature, with nitro reduction being the most likely metabolic process. The results of the electrochemical reduction of the studied API’s are summarized in Table 2, and proposed structures are presented in Figure 11, Figure 12, Figure 13 and Figure 14.

The HRMS analysis of dantrolene spectra after electrochemical reduction indicated the two degradation products, DAN-Imp1-RED and DAN-Imp2-RED, with molecular ion peaks at *m*/*z* 299.0785 [M−H]^−^ and *m*/*z* 282.0758 [M−H]^−^. Figures S13 and S14 display the MS2 spectra of these impurities, while Figures S30 and S31 present the structures of characteristic fragments, confirming their identification. MS spectra of the dantrolene sample, measured at negative potentials from 0 to −2 V, showed the formation of hydroxylamine, previously described in the literature [41,42], and the new reduction impurity.

The HRMS analysis of furazidine spectra after electrochemical reduction also indicated the reduction of nitro groups. The following three new degradation products were observed: FUR-Imp1-RED (at −0.7 V), FUR-Imp2-RED (at −0.7 V), and FUR-Imp3-RED (at −0.9 V), with molecular ion peaks at *m*/*z* 249.0657 [M−H]^−^, *m*/*z* 247.0476 [M−H]^−^, and *m*/*z* 233.0703 [M−H]^−^. Figures S15–S17 illustrate the MS2 spectra for these impurities, while Figures S32–S34 present the structures of characteristic fragments, confirming their identification.

In the case of nitrofurazone, the detection of two products with *m*/*z* 184.0363 and *m*/*z* 167.0572 in electrochemical reduction experiments led to the identification of an amine (NF-Imp1-RED), which is formed at a potential of −1.0 V and a hydroxylamine known from the literature (NF-Imp2-RED), which is formed at a potential of −0.7 V (Figures S18 and S19). Figures S35 and S36 present the structures of characteristic fragments, confirming their identification.

The formation of three degradation products was confirmed in the nitrofurantoin reduction studies. HR-MS spectra showed that the highest-intensity impurity, NFT-Imp1-RED, is an amine formed at a potential of −1.0 V, with *m*/*z* 207.0518. According to our study, the reduction of nitrofurantoin also involves the opening of the nitrofurane ring (−0.7 V), *m*/*z* 205.0366. At a reduction potential of −1.0 V, a hydroxylamine, NFT-Imp3-RED with *m*/*z* 223.0460, is also formed. Figures S20–S22 illustrate the MS2 spectra of nitrofurantoin’s impurities, while Figures S37–S39 present the structures of characteristic fragments, confirming their identification.

The electrochemical studies are consistent with the metabolism of nitroreductases reported in the literature.

A comparison of the reduction products obtained by the electrochemical method and the metabolites predicted in silico is summarized in Table 2.

### 2.3. Quantum Mechanical DFT Calculations

The optimized molecular structures were obtained with the Gaussian G16 software, Revision A.03 [49] using the density functional theory (DFT) with the B3LYP/6-311++G(d,p) function and optimization with the Berny algorithm until positive harmonic frequencies were obtained. The Gibbs Free Energy (in kcal/mol) was used as the metric for comparing the relative stabilities of the investigated structures (the ΔG values in Table 1 and Table 2). The details of the calculations are presented in the Supplementary Information, see the Tables S1 and S2.

An analysis of the Gibbs Free Energy differences assigned to the hypothetical stoichiometric reaction leads to the following observations.

#### 2.3.1. Oxidation

Oxidation seems to be a regioselective addition of mono or dioxygen addition to the molecular structure. The most preferred position for the mono-oxygen addition is the -CH- residue between the nitrogen atom and the furan ring. Subsequent oxygen additions occur at various positions, such as other -CH- or NH- residues. The following observations of the ΔG values can be made:

For NFT, the monooxidation of NFT is resolved to be the structure NFT-Imp1-Ox with a ΔG = −47 kcal/mol. The dioxidation of NFT is identified as the structure NFT-Imp3-Ox and corresponds well with a ΔG = −100 kcal/mol, i.e., the most negative value among ΔG values of other di-oxygenated structures.

The monooxidation of DAN is resolved to be the structure DAN-Imp1-Ox, and its ΔG = −52 kcal/mol is the most negative value among the two monooxidated structures investigated theoretically. The dioxidation of DAN occurs at the sites observed in the monooxidation, i.e., at the -CH_2_- residue of the imidazolidinedione ring and at the -CH=N- fragment of the hydrazone linker.

The monooxidation of NF is resolved to be the structure NF-Imp1-Ox, and its ΔG = −54 kcal/mol is the most negative value among the three monooxidated structures investigated theoretically.

The monooxidation of FUR is resolved to be the structure FUR-Imp1-Ox based on two structural proposals. The second structural proposal corresponds to ΔG = −9 kcal/mol, also known as the less energetically favorable among the five structures considered theoretically. The most energy advantageous is the first structural proposal, for which the energy is ΔG= −47 kcal/mol.

Compounds oxidize either at the carbon in the aliphatic ring or in the five-membered ring at the carbon. Whereby, in DAN, the first oxidation goes to the carbon in the aliphate and the second to the ring. In NF, the first oxidation also goes to the carbon in the aliphatic chain. There is no second oxidation. One can comment on this effect, i.e., the double oxidation of DAN and monooxidation of NF, with a comparison of the hypothetical stoichiometric reactions, and the corresponding Free Energy output (ΔG) can be predicted theoretically as follows:

DAN + ^1^/_2_ O_2_ → DAN-Imp1-Ox(A) (monooxidation) ΔG = −52 kcal/mol

DAN + O_2_ → DAN-Imp2-Ox (dioxidation) ΔG = −98 kcal/mol

The monooxidation of DAN occurs at the aliphatic residue. In the dioxidation process, the second oxygen atom is located at the imidazolidine-2,4-dione residue. The negative ΔG values suggest that both reactions should occur spontaneously. In the case of NF, only monooxidation is observed. The corresponding hypothetical reaction is as follows:

NF + ^1^/_2_ O_2_ → NF-Imp1-Ox (monooxidation) ΔG = −54 kcal/mol

However, dioxidation should not occur due to unfavorable ΔG values for the oxygen atom insertion into the imidazolidine-2,4-dione residue of ΔG = −18 kcal/mol (at the imino nitrogen) and ΔG = −7 kcal/mol (at the amino nitrogen). Taking the above observations into account, one can elucidate the reason for the lack of double oxidation in NF, and consequently, its greater resistance to oxidation compared to DAN, which should be less resistant. According to the scheme for FUR, we should consider two monooxidated structures of FUR, i.e., FUR-Imp1-Ox with the OH group attached to the carbon atom of the -CH_2_- residue and to the nitrogen atom located between two oxygen atoms. According to the calculated ΔG values (−51 kcal/mol and −9 kcal/mol, respectively), the second structure is less likely.

#### 2.3.2. Reduction

The reduction reactions seem to follow a variety of paths, as a rule leading to a degradation of the parental (initial) molecule. The stoichiometric reactions suggest that, in the reduction process, 1–6 electrons are exchanged. The stoichiometric reaction does not incorporate details of the molecular mechanism of the reduction reaction. One can only expect that the more electrons that are exchanged, the more complex the reaction, e.g., multistep reactions, should occur. A tentative generalization based on our empirical and theoretical data suggests preserving the entire structure of the parent molecule. An exception is the case of the NFT-Imp2-Ox structure, where NFT oxidation results in the opening of the imidazolidinedione ring. This may provide another argument supporting our belief in the low stability of the parent NFT molecule. In contrast to oxidation, the reduction reaction leads to the degradation of the parent molecules, splitting them into various smaller fragments.

An analysis of the hypothetical ΔG (reaction) is shown as follows. Let us schematically describe the molecular structure of the parent molecules as follows:

NF (nitrofural): (nitrofuran residue) – (-C=N-) – (formamide residue)

NFT (nitrofurantoin): (nitrofuran residue) – (-C=N-) – (imidazolidinedione residue)

FUR (furazidine): (nitrofuran residue) – (-C=C-C=N-) – (imidazolidinedione residue)

DAN (dantrolene): (nitrophenyl-nitrofuran residue) – (-C=N-) – (imidazolidinedione residue).

The degradation of the parent molecules occurs at each molecular fragment described above, depending on the experimental conditions and a particular molecular structure of the parent molecule. The manner in which specific degradation under reductive conditions occurs can be interpreted with the theoretically calculated ΔG (reaction).

The estimated ΔG (reaction) of a series of stechiometric hypothetical reductive reactions falls within the [−400, −11] kcal/mol interval. We believe that the more negative the ΔG, the more likely the corresponding degradation of the parent molecules. Following this reasoning, we predict that, based on the ΔG (reaction) in the [−400, −300] kcal/mol range, one should expect the following degradation paths:

- NF (nitrofural) undergoes first the NO_2_- group reduction up to the amino -NH_2_ group.
- NFT (nitrofurantoin): here, reduction of the NO_2_ group to the -NH_2_ group becomes the most probable degradation path.
- FUR (furadizine): here too, the reduction of the NO_2_ group to the -NH_2_ group becomes the most probable degradation path.
- DAN (dantrolene): the degradation of the NO_2_ group to -NH-OH or =NH residues becomes the most likely path. Here, the reduction does not terminate at the -NH_2_ group.

It is suggested that these paths correspond to the exchange of six electrons. It is also expected that the molecular mechanism of such degradation is much more complicated than that visualized in a simple six-electron hypothetical stoichiometric reaction.

The reduction of the DAN, FUR, NF, and NFT compounds generally yields amino- or hydroxylamino derivatives (the -NO_2_ residue reduction). Moreover, in one of the NFT derivatives, degradation results in the opening of the furan ring and the formation of the CN triple bond.

By reviewing the ΔG values of the corresponding hypothetical reduction reactions, one cannot identify any outliers because all the investigated structures have similar ΔG values, of about −50 kcal/mol per one hydrogen atom added. Details are given in the Supplementary Materials, Table S1 and S2.

## 3. Materials and Methods

### 3.1. Chemicals

Acetonitrile for LC/MS, methanol for LC/MS, ammonium formate for LC/MS, ammonia solution, formic acid for LC/MS, and dimethyl sulfoxide were purchased from Merck (Darmstadt, Germany).

### 3.2. Electrochemical Measurements

Electrochemical studies were performed using an Autolab potentiostat (Eco Chemie BV, Utrecht, The Netherlands), which was controlled by a computer with GPES software, version 4.9. A three-electrode system placed in a glass measuring vessel was used in these studies. This system consisted of a reference electrode, a saturated silver/silver chloride electrode with saturated KCl (Ag/AgCl, KCl_sat_), a platinum plate, which acted as an auxiliary electrode, and a working electrode, a glassy carbon electrode with a surface area of 0.071 cm^2^ (GCE, BASi^®^, Kent Avenue, West Lafayette, IN, USA). The solutions in which all three electrodes were immersed were deoxygenated by passing argon (99.5% purity, Air Products, Kielce, Poland) for 20 min before the measurements. During the measurements, gas was passed over the solution. Measurements were carried out at an ambient temperature of approximately 22 ± 2 °C.

### 3.3. EC/ESI-HRMS Measurements

Electrochemical oxidation and reduction were performed using the ROXY^TM^ EC system (AntecScientific, Zoeterwoude, The Netherlands) [50]. The ROXY™ EC System (Figure S40) includes the ROXY potentiostat equipped with a µ-PrepCell™ 2.0, infusion pump (HARVARD APPARATUS, Holliston, MA, USA), and a glass syringe (5.0 mL, HAMILTON Co., Reno, NV, USA). The ROXY EC System is controlled by Antec Dialogue software, version 2.0.0.81. The µ-PrepCell™ 2.0, equipped with a Magic Damond (BBD) working electrode, an auxiliary (AUX) electrode (inert polymeric inlet block), and a HyREF™ reference electrode, was used for the generation of degradation and metabolism impurities. The experiments were operated at a flow rate of 50 µL/min at room temperature and a potential range for oxidation from 0 to 2.0 V and for reduction from 0 to −2.0 V. DAN, FUR, NF, and NFT (Figure 2) were dissolved in 20 mM of aqueous ammonium formate solution (pH = 7.4) and ACN (50:50 *v*/*v*). The final concentration of APIs was 100 µg/mL.

The EC cell was connected to the ESI-MS-HR: Maxis 4G time-of -flight mass analyzer (Bruker, Bremen, Germany). The QTOF system was controlled by MAXIS software, Compass 1.3, control version 3.0 and data processing DataAnalysis software, version 4.0 SP3. The HR-MS runs were performed in positive or negative mode depending on the API used with the following parameters: nebulizer 0.4 bar, dry gas 4 L/min, dry temperature 180 °C, capillary +2800 V, and end plate −500 V. For EC/HRMS coupling, a mass range from 50 to 1000 *m*/*z* was used. The mass spectrometer was tuned before any measurements, and sum formulas were calculated for the resulting measured ion masses. The molecular patterns were determined with a high degree of precision, keeping the mass error below 5 ppm and the isotopic pattern deviation, known as mSigma, below 30. The mSigma value is a measure of how well the theoretical isotopic pattern matches the observed pattern for a given mass peak. It includes the standard deviation of both masses and intensities of all isotopic peaks. For the hybrid mass spectrometer, the precursor ion of the tested compound was selected using the quadrupole analyzer, and the product ions were analyzed using a TOF analyzer.

Structure elucidation was facilitated using ACD/Labs MS Fragmenter software, version 2023.2.0.

### 3.4. DFT Calculations

All the calculations were performed on the HPC cluster in the Interdisciplinary Centre for Mathematical and Computational Modelling at the University of Warsaw, Poland.

The model reactions of the reduction/oxidation (red/ox) processes were assumed to proceed from a known initial step (Reactants) up to the deduced final step (Products) without considering any intermediate transition structures or reactions. In this way, we assumed that the model reaction can proceed spontaneously when the difference between the Product Gibbs Free Energy and the Reactant Gibbs Free Energy becomes negative.

### 3.5. In Silico Prediction of Metabolism

In silico predictions were performed using Bruker Daltonics software (version 2.0; Germany). The MetabolitePredict (version 2.0; Bruker Daltonics, Bremen, Germany) is a computational algorithm that predicts the most likely metabolic transformations of the compound related to phase I reactions in the liver. Moreover, it provides the structure of the metabolites, formed with a ranking derived from the site of metabolism predictions, with exact molecular weight (MW).

## 4. Conclusions

Pharmaceutical compounds such as dantrolene, nitrofurantoin, furazidine, and nitrofural were investigated electrochemically to determine their electroactivity and to generate degradation products. Oxidation and reduction products were predicted and supported by a computational approach that included an in silico method and a quantum mechanical DFT calculation of their molecular energies. Seventeen degradation impurities were identified using the QTOF technique, and most of these products were also confirmed by computational methods. The electrochemical technique has generated a large number of structurally related products and has proven effective in producing new, not previously reported, degradative impurities, the toxicity of which is worth considering. Additionally, electrochemistry allows us to understand the stability of a drug product with hydrazone groups. In electrochemical oxidation studies, nitrofurantoin proved to be the most unstable, forming degradable impurities at a potential from +0.7 V, while furazidine proved to be the most stable, with an oxidation product formed at +2.0 V. For all hydrazone-containing drugs tested, the stability of reducing potentials was similar, with degradation impurities formed from −0.7 V. Examination of the stability towards oxidation of the carbon neighboring the hydrazone group indicates that compounds such as dantrolene and nitrofurantoin may be more susceptible to the hydrolysis of the hydrazone and the formation of amines or hydroxyamines, which can be oxidized to nitrosamines under the right conditions. The present research becomes essential in the design of methodology applied to protecting patient safety and developing robust products. The research conducted also determines the conditions under which electrochemical synthesis of the newly identified impurities will be possible. This work continues to focus on the detection of low-molecular-weight nitrosamines.

## Figures and Tables

**Table 1:** Summary of the main oxidation product of four tested APIs.

Compound	MW	Molecular Formula	Initial Conditions	Suggested Transformation	EC	In Silico	DFT Gibbs Energy [kcal/mol]
**Dantrolene (DAN)**	**314.253**	**C_14_H_10_N_4_O_5_**	-	-	-	-	-
**Dantrolene impurities**							
DAN-Imp1-Ox(A) DAN-Imp1-Ox(B)	330.252	C_14_H_10_N_4_O_6_	pH 7.4, +1.0	+O	√ X	X √	−52 −47
DAN-Imp2-Ox	346.252	C_14_H_10_N_4_O_7_	pH 7.4, +1.2 V	+2O	√	X	−98
**Furazidine (FUR)**	**264.194**	**C_10_H_8_N_4_O_5_**	-	-	-	-	-
**Furazidine impurities**							
FUR-Imp1-Ox(A) FUR-Imp1-Ox(B)	280.194	C_10_H_8_N_4_O_6_	pH 7.4, +2.0 V	+O	X √	√ X	−47 −9
**Nitrofural (NF)**	**198.136**	**C_6_H_6_N_4_O_4_**	-	-	-	-	-
**Nitrofural impurities**							
NF-Imp1-Ox	214.136	C_6_H_5_N_4_O_5_	pH 7.4, +1.5	+O	√	X	−54
**Nitrofurantoin (NFT)**	**238.157**	**C_8_H_6_N_4_O_5_**	-	-	-		-
**Nitrofurantoin impurities**							
NFT-Imp1-Ox	254.156	C_8_H_6_N_4_O_6_	pH 7.4, +0.8 V	+O	√	√	−47
NFT-Imp2-Ox	225.158	C_8_H_7_N_3_O_5_	pH 7.4, +1.0 V	-N+H	√	X	−190
NFT-Imp3-Ox	270.156	C_8_H_6_N_4_O_7_	pH 7.4, +1.2 V	+2O	√	X	−100

EC—electrochemistry; MetabolitePredict—in silico; MW—molecular weight.

**Table 2:** Summary of the main reduction products of four tested APIs.

Compound	MW	Molecular Formula	Conditions	Suggested Transformation	EC	In Silico	DFT Gibbs Energy [kcal/mol]
**Dantrolene (DAN)**	**314.253**	**C_14_H_10_N_4_O_5_**	-	-	-	-	-
**Dantrolene impurities**							
DAN-Imp1-RED	300.270	C_14_H_12_N_4_O_4_	pH 7.4, −0.7 V	+2H -O	√	√	−239
DAN-Imp2-RED	282.259	C_14_H_10_N_4_O_3_	pH 7.4, −0.7 V	-2O	√	X	−204
**Furazidine (FUR)**	**264.194**	**C_10_H_8_N_4_O_5_**	-	-	-	-	-
**Furazidine impurities**							
FUR-Imp1-RED	234.211	C_10_H_10_N_4_O_3_	pH 7.4, −0.7 V pH 7.4, −2.0 V	+2H -2O	√	√	−398
FUR-Imp2-RED	248.195	C_10_H_8_N_4_O_4_	pH 7.4, −0.7 V	-O	√	X	−128
FUR-Imp3-RED	250.211	C_10_H_10_N_4_O_4_	pH 7.4, −0.9 V	+2H -O	√	√	−242
**Nitrofural (NF)**	**198.136**	**C_6_H_6_N_4_O_4_**	-	-	-	-	-
**Nitrofural impurities**							
NF-Imp1-RED	168.153	C_6_H_8_N_4_O_2_	pH 7.4, −1.0 V	+2H -2O	√	√	−399
NF-Imp2-RED	185.137	C_6_H_7_N_3_O_4_	pH 7.4, −0.7 V	+H -N	√	X	−179
**Nitrofurantoin (NFT)**	**238.157**	**C_8_H_6_N_4_O_5_**	-	-	-	-	-
**Nitrofurantoin impurities**							
NFT-Imp1-RED	208.174	C_8_H_8_N_4_O_3_	pH 7.4, −1.0 V	+2H -2O	√	√	−400
NFT-Imp2-RED	206.158	C_8_H_6_N_4_O_3_	pH 7.4, −0.7 V	-2O	√	X	−286
NFT-Imp3-RED	224.174	C_8_H_8_N_4_O_4_	pH 7.4, −1.0 V	+2H -O	√	√	−244

EC—electrochemistry; MetabolitePredict—in silico; MW—molecular weight.

## Data Availability

The original contributions presented in this study are included in the article and Supplementary Materials. Further inquiries can be directed to the corresponding author.

## Supplementary Materials

The supporting information can be downloaded at the following: https://www.mdpi.com/article/10.3390/ijms26094295/s1.
