# Supplementary material for: Oxidation and Reduction of Hydrazones—Risk Factors Related to the Manufacture and Stability of the Drugs
Source: Int J Mol Sci. 2025 May 1;26(9):4295. doi: 10.3390/ijms26094295 (PMC12072505; doi:10.3390/ijms26094295)
Supplement: Supplementary file 1 [file ijms-26-04295-s001.zip › ijms-3587846-supplementary.pdf]

Oxidation and reduction of hydrazones-risk factors related to the manufacture and stability of the drugs

**Anna B. Witkowska[1,2], Krzysztof Stolarczyk[3], Massimo Fusaro[3], Andrzej Leś[3], Joanna Giebułtowicz[2], and Elżbieta U. Stolarczyk[1]*

[1] Spectrometric Methods Department, National Medicines Institute, Chełmska 30/34, 00-725 Warsaw, Poland, e.stolarczyk@nil.gov.pl (E.U.S.), a.witkowska@nil.gov.pl (A.B.W.)
[2] Department of Drug Chemistry, Medical University of Warsaw, 61 Żwirki i Wigury, 02-091 Warsaw, Poland; j.giebułtowicz@wum.edu.pl
[3] Faculty of Chemistry, University of Warsaw, 1 Pasteura Street, 02-093 Warsaw, Poland; kstolar@chem.uw.edu.pl (K.S.), ales@chem.uw.edu.pl (A.L.), maxitp@gmail.com (M.F.).
* Correspondence: e.stolarczyk@nil.gov.pl

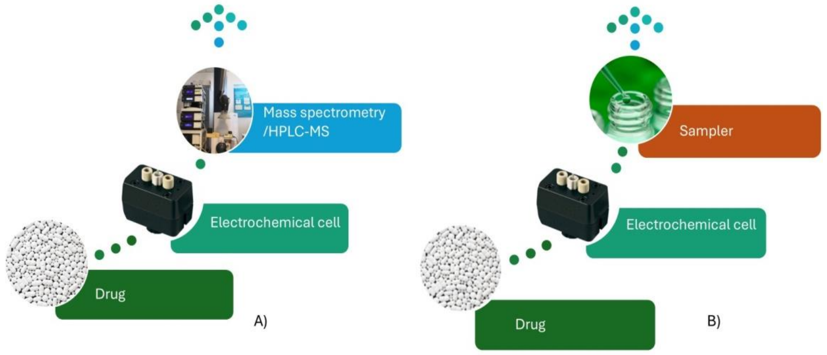

Fig. S1. Configuration for coupling electrochemistry and mass spectrometry: A) on-line EC/MS mode, B) off-line EC mode.

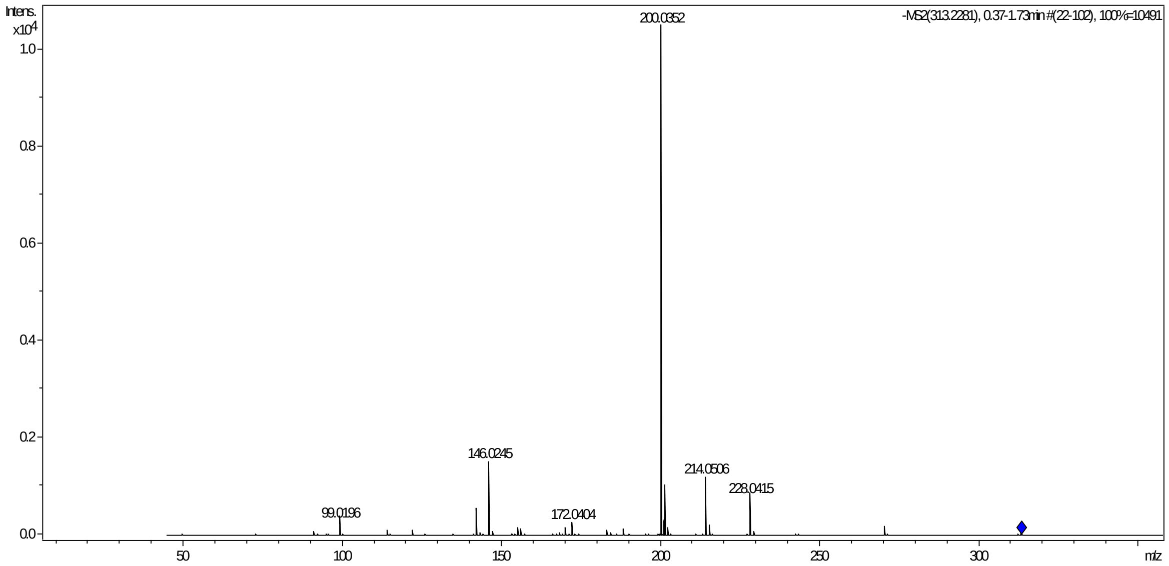

Fig. S2. High-resolution mass spectrometry MS^2 spectrum of DAN in negative ionization mode.

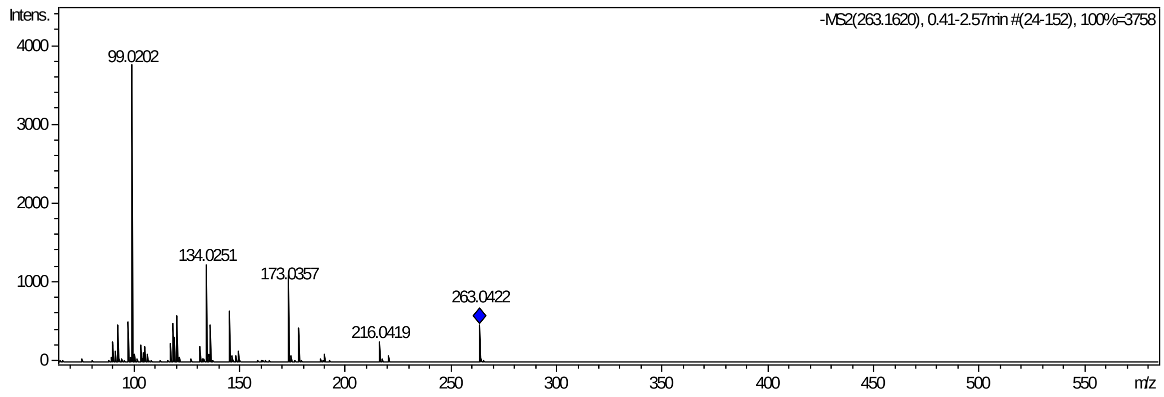

Fig. S3. High-resolution mass spectrometry MS^2 spectrum of FUR in negative ionization mode.

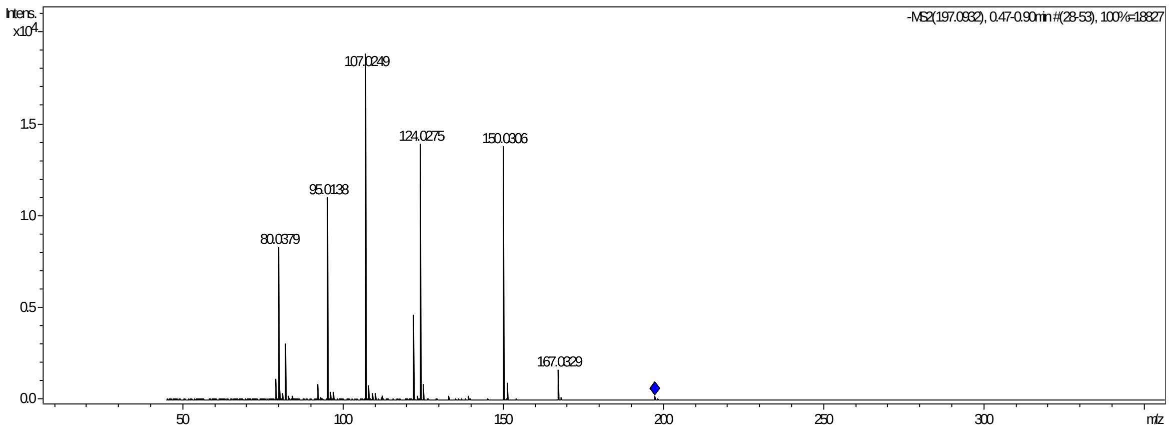

Fig. S4. High-resolution mass spectrometry MS^2 spectrum of NF in negative ionization mode.

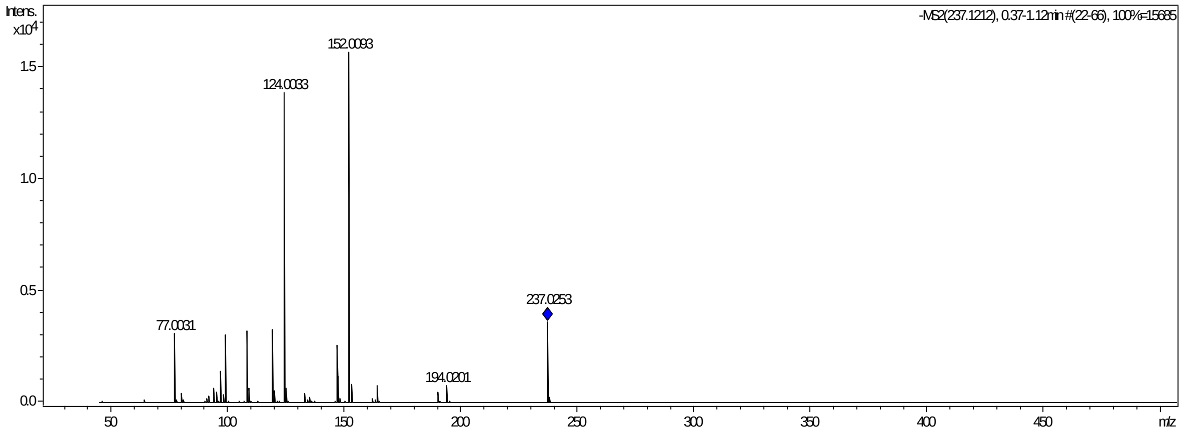

Fig. S5. High-resolution mass spectrometry MS^2 spectrum of NFT in negative ionization mode.

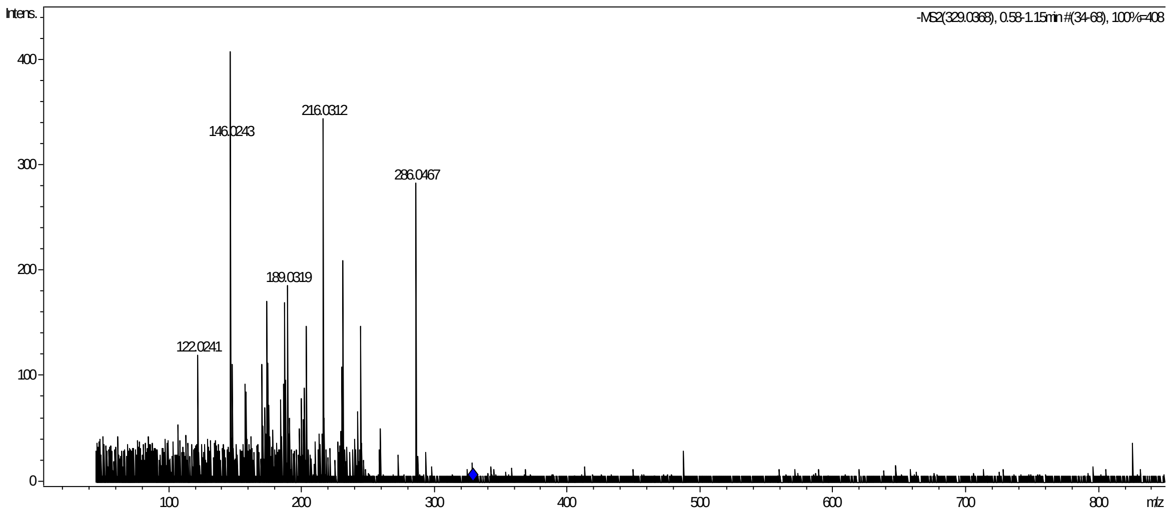

Fig. S6. High-resolution mass spectrometry MS^2 spectrum of DAN product degradation – DAN-Imp1-Ox resulting from oxidation in negative ionization mode.

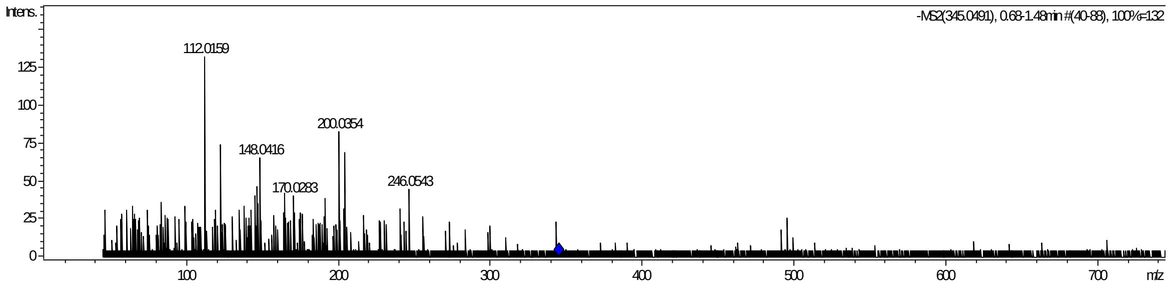

Fig. S7. High-resolution mass spectrometry MS^2 spectrum of DAN product degradation – DAN-Imp2-Ox resulting from oxidation in negative ionization mode.

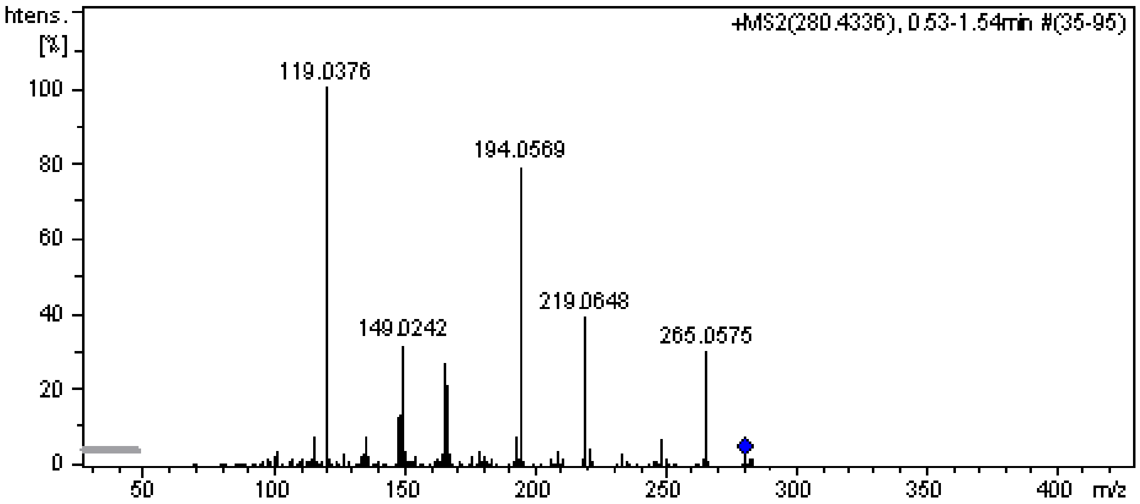

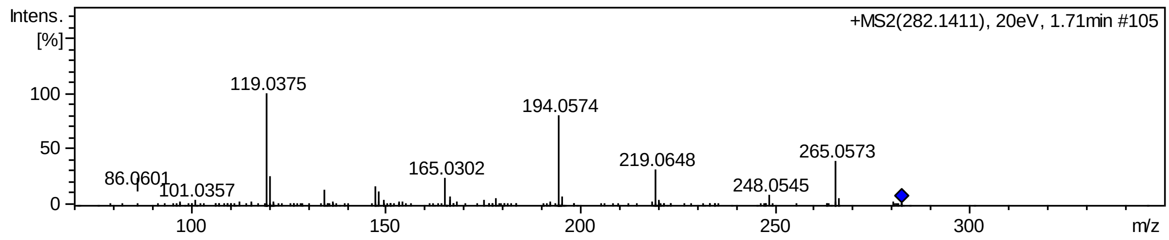

Fig. S8. High-resolution mass spectrometry MS[2] spectrum of FUR product degradation – FUR-Imp1-Ox resulting from oxidation in positive ionization mode.

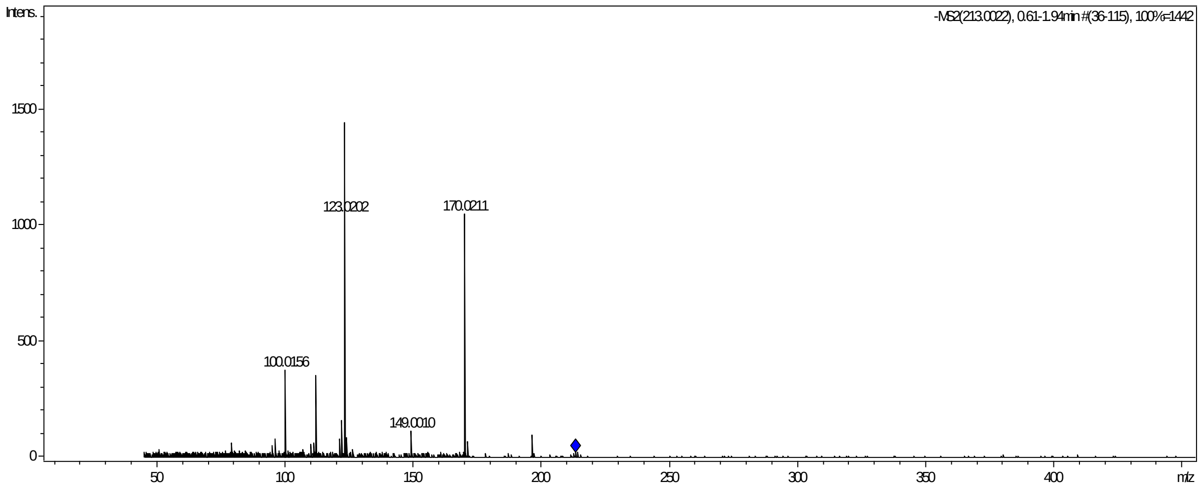

Fig. S9. High-resolution mass spectrometry MS[2] spectrum of NF product degradation – NF-Imp1-Ox resulting from oxidation in negative ionization mode.

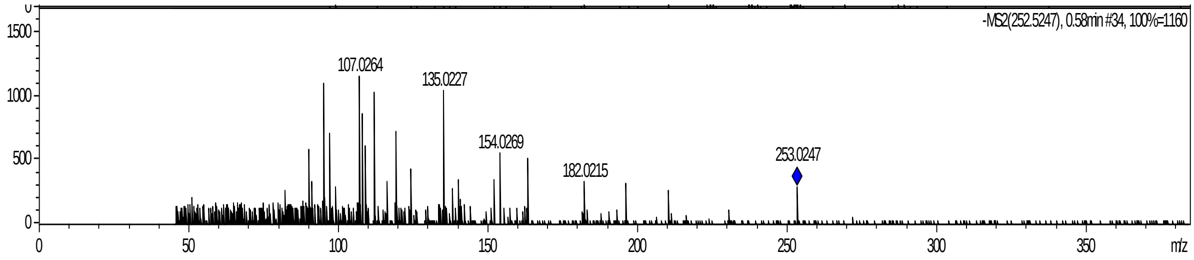

Fig. S10. High-resolution mass spectrometry MS[2] spectrum of NFT product degradation – NFT-Imp1-Ox resulting from oxidation in negative ionization mode.

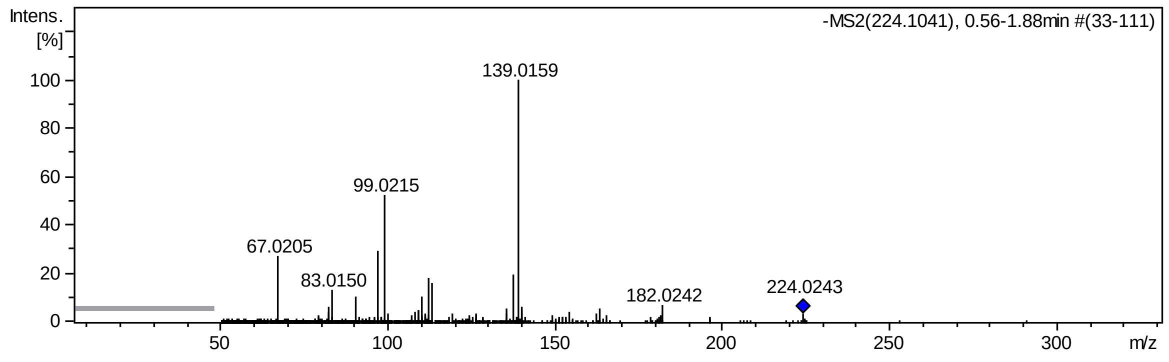

Fig. S11. High-resolution mass spectrometry MS^2 spectrum of NFT product degradation – NFT-Imp2-Ox resulting from oxidation in negative ionization mode.

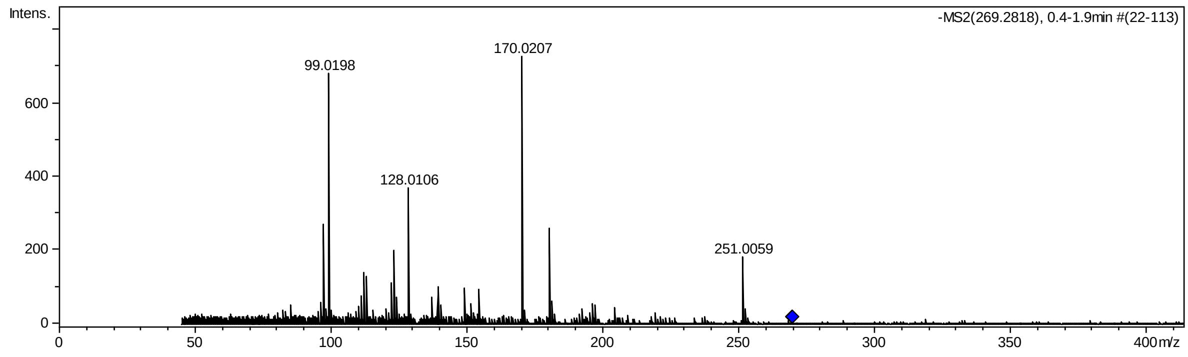

Fig. S12. High-resolution mass spectrometry MS^2 spectrum of NFT product degradation – NFT-Imp3-Ox resulting from oxidation in negative ionization mode.

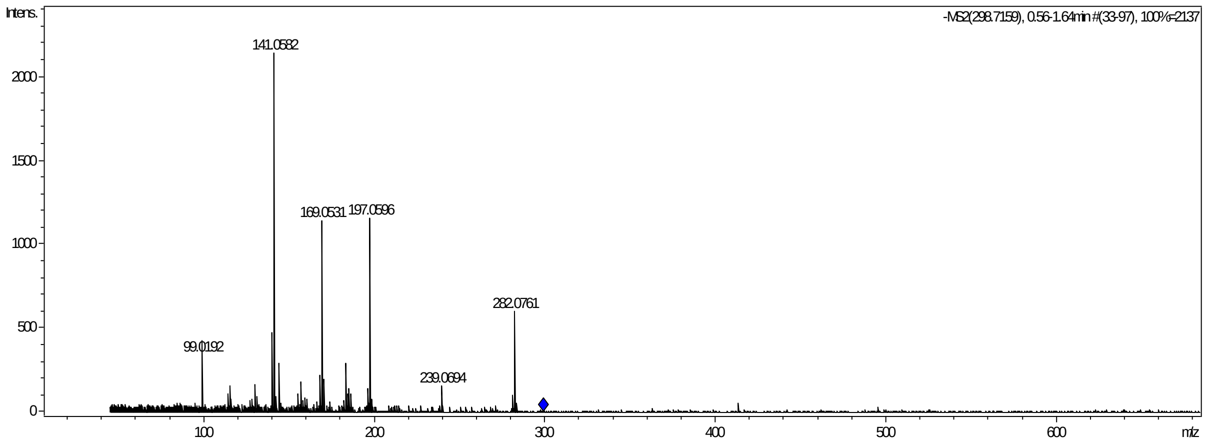

Fig. S13. High-resolution mass spectrometry MS^2 spectrum of DAN product degradation – DAN-Imp1-RED resulting from reduction in negative ionization mode.

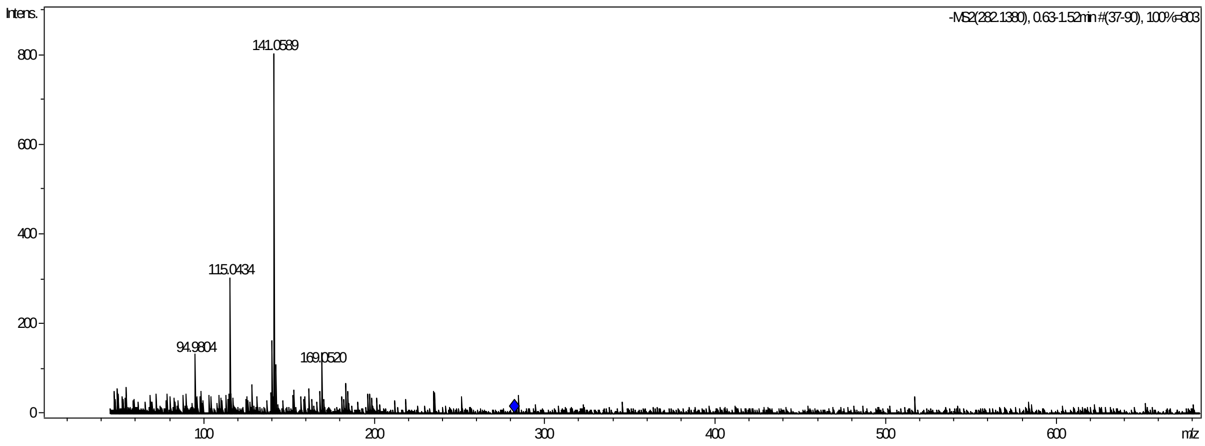

Fig. S14. High-resolution mass spectrometry MS² spectrum of DAN product degradation – DAN-Imp2-RED resulting from reduction in negative ionization mode.

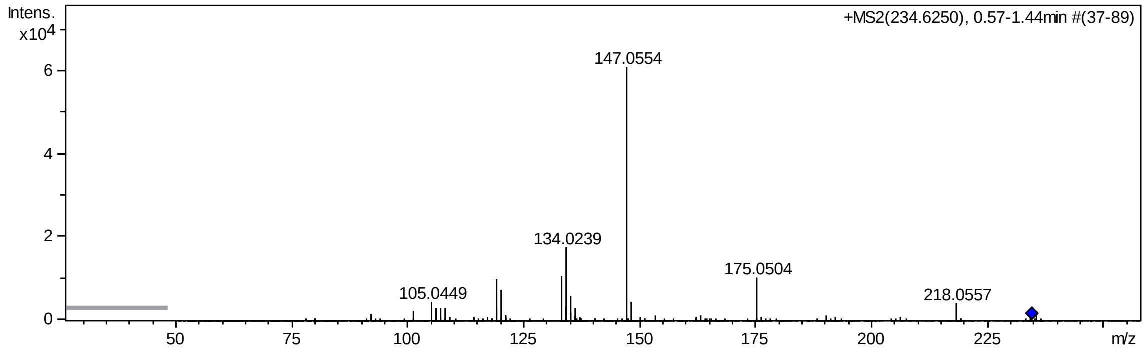

Fig. S15. High-resolution mass spectrometry MS² spectrum of FUR product degradation – FUR-Imp1-RED resulting from reduction in positive ionization mode.

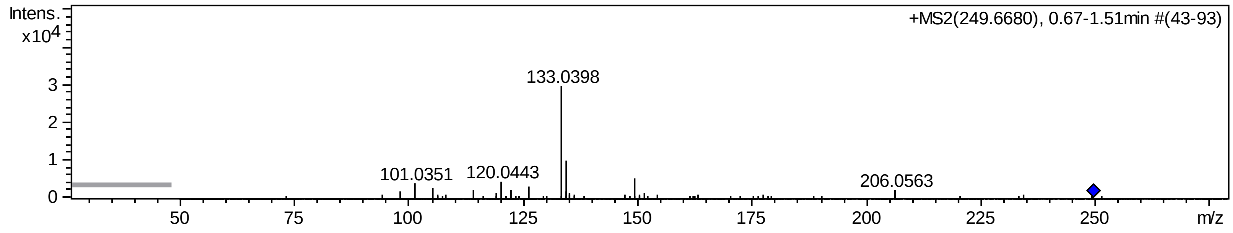

Fig. S16. High-resolution mass spectrometry MS² spectrum of FUR product degradation – FUR-Imp2-RED resulting from reduction in positive ionization mode.

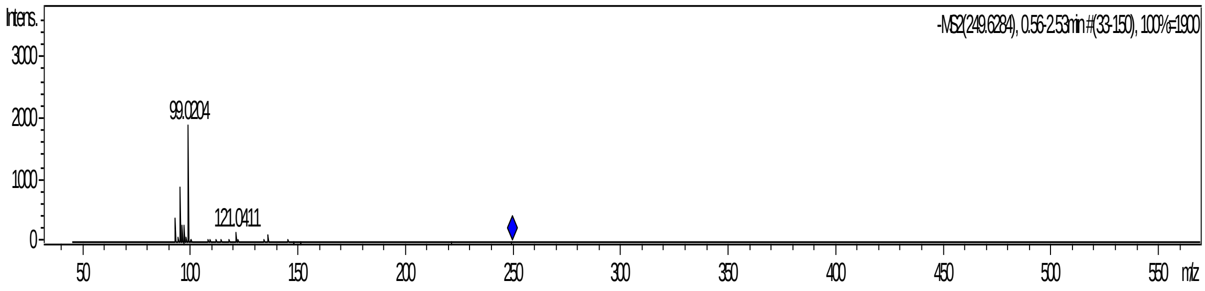

Fig. S17. High-resolution mass spectrometry MS² spectrum of FUR product degradation – FUR-Imp3-RED resulting from reduction in negative ionization mode.

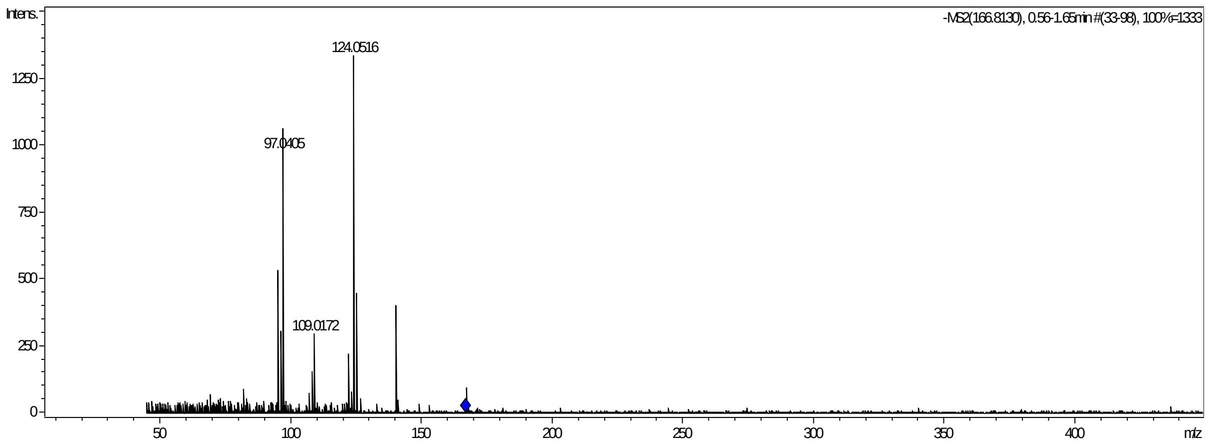

Fig. S18. High-resolution mass spectrometry MS² spectrum of NF product degradation – NF-Imp1-RED resulting from reduction in negative ionization mode.

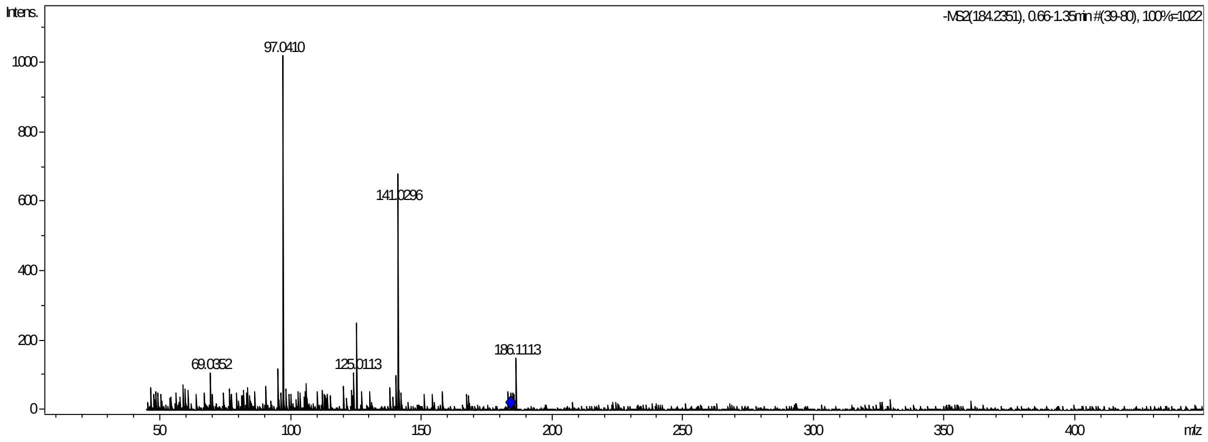

Fig. S19. High-resolution mass spectrometry MS² spectrum of NF product degradation – NF-Imp2-RED resulting from oxidation in negative ionization mode.

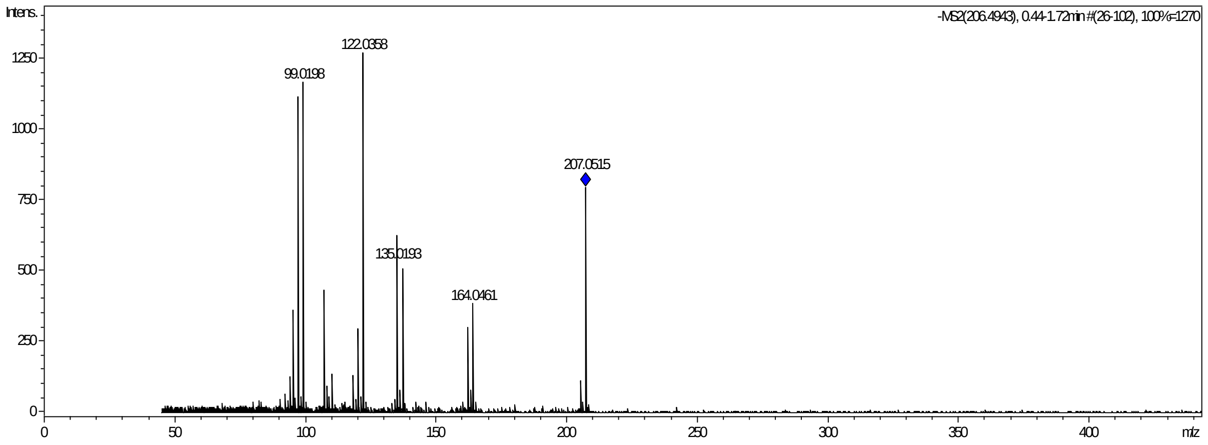

Fig. S20. High-resolution mass spectrometry MS² spectrum of NFT product degradation – NFT-Imp1-RED resulting from reduction in negative ionization mode.

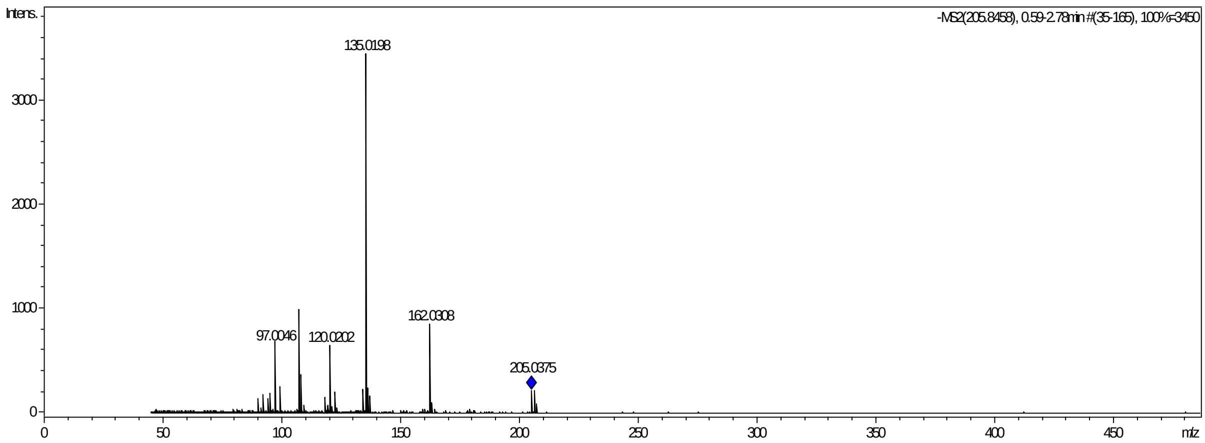

Fig. S21. High-resolution mass spectrometry MS^2 spectrum of NFT product degradation – NFT-Imp2-RED resulting from reduction in negative ionization mode.

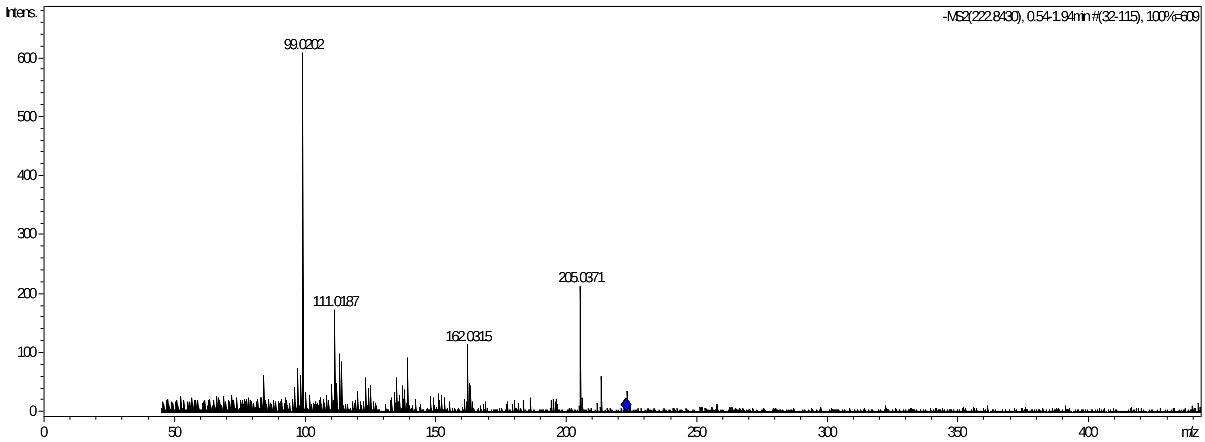

Fig. S22. High-resolution mass spectrometry MS^2 spectrum of NFT product degradation – NFT-Imp3-RED resulting from reduction in negative ionization mode.

Dantrolene (DAN):

DAN-Imp1-OX

Molecular Formula: $C_{14}H_{10}N_4O_6$

Formula Weight: 330.2524

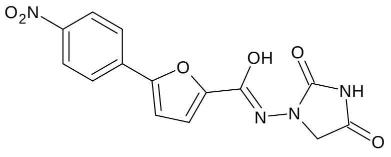

$C_{13}H_8N_3O_5$; m/z 286:

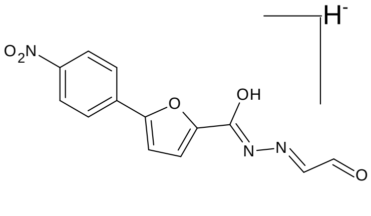

$C_{11}H_6NO_4$; m/z 216:

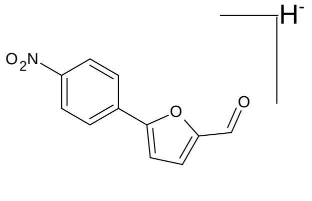

$C_{11}H_7N_2O_4$; m/z 231:

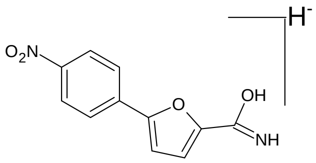

$C_9H_5N_2O_3$; m/z 189:

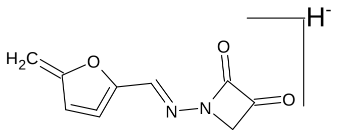

$C_8H_4NO_2$; m/z 146:

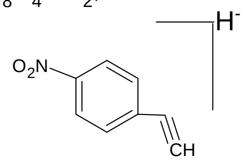

$C_6H_4NO_2$; m/z 122:

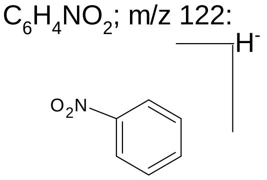

Fig. S23. The proposed molecular structures of characteristic ions for DAN-Imp1-Ox.

Dantrolene (DAN):

DAN-Imp2-OX

Molecular Formula: $C_{14}H_{10}N_4O_7$

Formula Weight: 346.2518

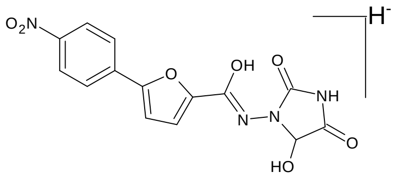

$C_{11}H_8N_3O_4$; m/z 246:

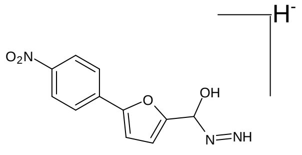

$C_{11}H_6NO_3$; m/z :200

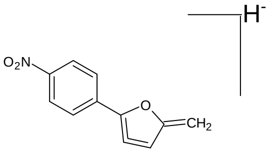

$C_8H_6NO_2$; m/z 148:

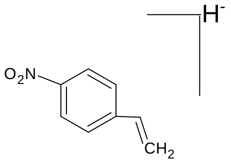

$C_6H_4NO_2$; m/z 122:

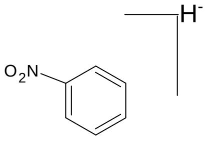

$C_3H_2N_3O_2$; m/z 112:

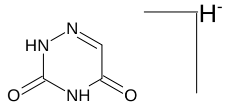

Fig. S24. The proposed molecular structures of characteristic ions for DAN-Imp2-Ox.

Furazidine (FUR):

FUR-Imp1-OX (B):

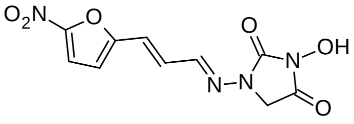

Molecular Formula: $C_{10}H_8N_4O_6$
Formula Weight: 280.19372

$C_{10}H_9N_4O_5$; m/z 265:

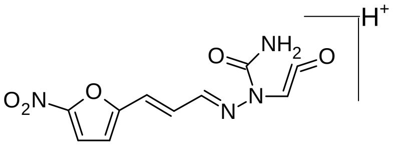

$C_9H_7N_4O_3$; m/z 219:

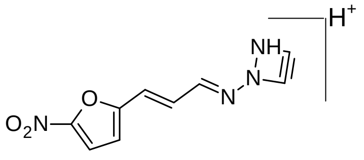

$C_{10}H_{10}N_3O_3$; m/z 220:

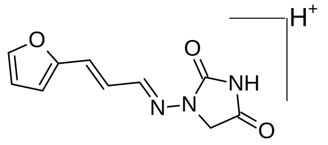

$C_8H_8N_3O_3$; m/z 194:

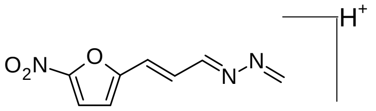

$C_8H_8N_3O_2$; m/z 192:

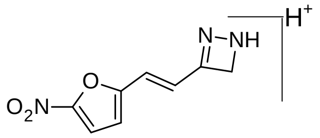

$C_7H_5N_2O_3$; m/z 165:

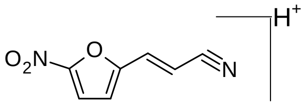

$C_7H_6N_3O$; m/z 148:

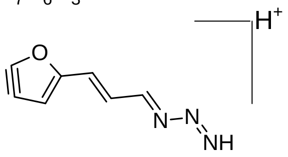

$C_8H_7N_2O$; m/z 147:

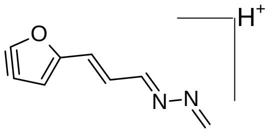

$C_7H_7N_2O$; m/z 135:

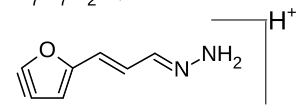

$C_7H_7N_2$; m/z 119:

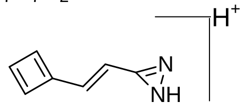

$C_3H_5N_2O_3$; m/z 117:

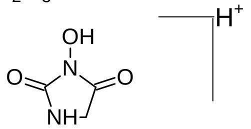

$C_3H_3N_2O_3$; m/z 115:

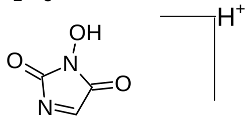

Fig. S25. The proposed molecular structures of characteristic ions for FUR-Imp1-Ox (A).

Nitrofural (NF):

NF-Imp1-OX

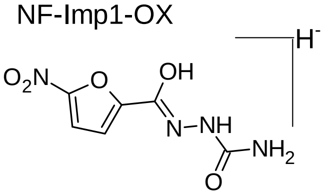

Molecular Formula: $C_6H_6N_4O_5$
Formula Weight: 214.13564

$C_6H_2N_3O_5$; m/z 196:

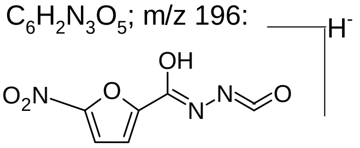

$C_5H_4N_3O_4$; m/z 170:

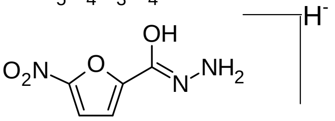

$C_6HN_2O_3$; m/z 149:

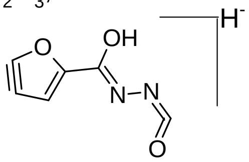

$C_5H_3N_2O_2$; m/z 123:

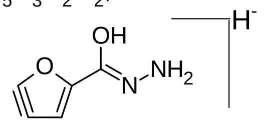

$C_2H_2N_3O_2$; m/z 100:

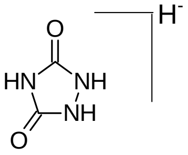

Fig. S26. The proposed molecular structures of characteristic ions for NF-Imp1-Ox.

Nitrofurantoin (NFT):

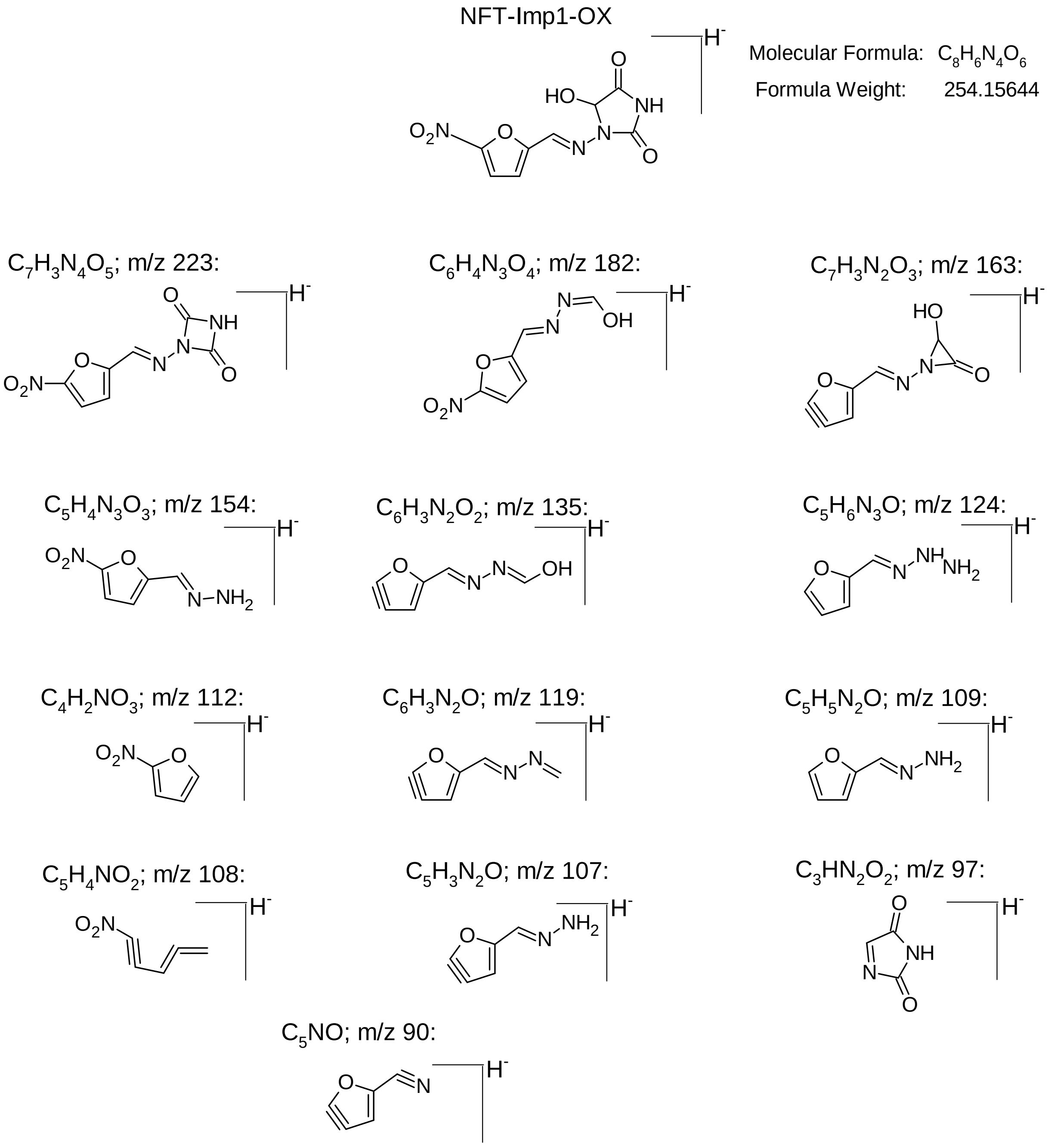

Fig. S27. The proposed molecular structures of characteristic ions for NFT-Imp1-Ox.

Nitrofurantoin (NFT):

NFT-Imp2-OX

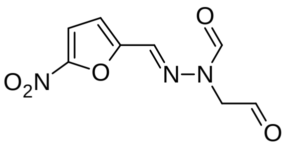

Molecular Formula: $C_8H_7N_3O_5$
Formula Weight: 225.15828

$C_6H_4N_3O_4$; m/z 182:

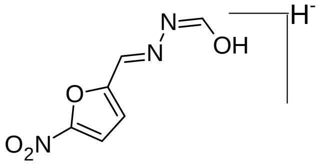

$C_5H_3N_2O_3$; m/z 139:

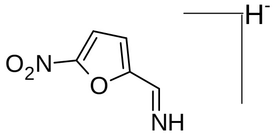

$C_3H_3N_2O_2$; m/z 99:

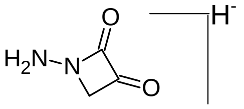

$C_3HN_2O_2$; m/z 97:

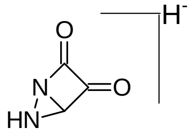

C_5NO; m/z 90:

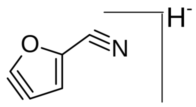

C_4H_3O; m/z 67:

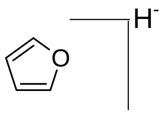

Fig. S28. The proposed molecular structures of characteristic ions for NFT-Imp2-Ox.

Nitrofurantoin (NFT):

NFT-Imp3-OX

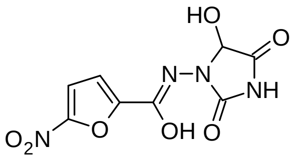

Molecular Formula: $C_8H_6N_4O_7$
Formula Weight: 270.15584

$C_8H_3N_4O_6$; m/z 251:

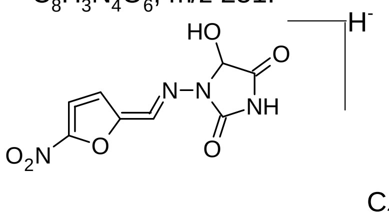

$C_5H_4N_3O_4$; m/z 170:

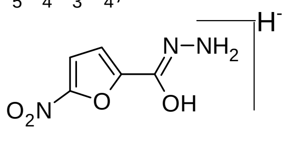

$C_3H_2N_3O_3$; m/z 128:

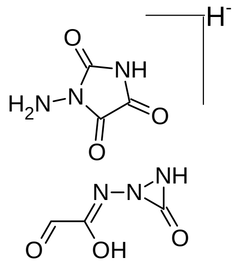

$C_3H_3N_2O_2$; m/z 99:

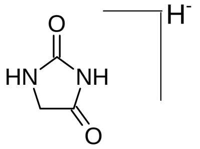

Fig. S29. The proposed molecular structures of characteristic ions for NFT-Imp3-Ox.

Dantrolene (DAN):

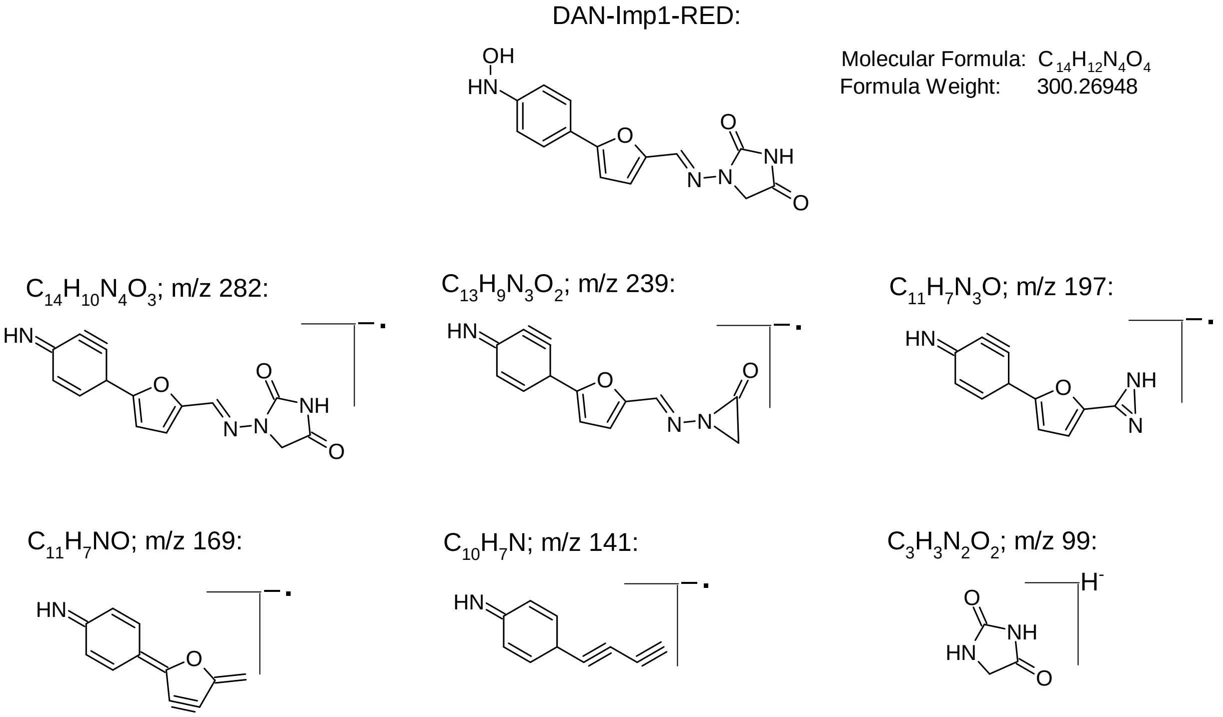

Fig. S30. The proposed molecular structures of characteristic ions for DAN-Imp1-RED.

Dantrolene (DAN):

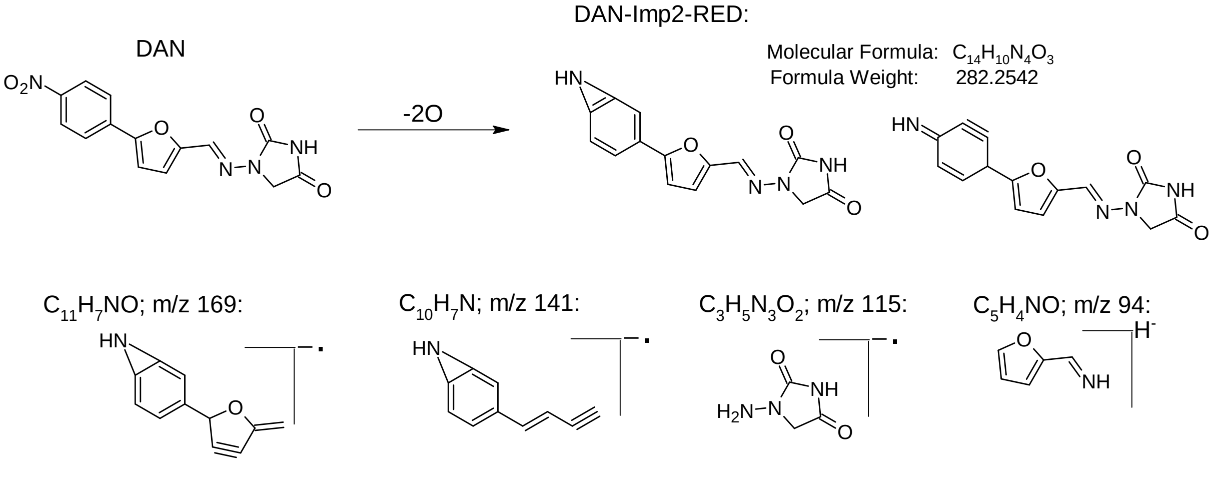

Fig. S31. The proposed molecular structures of characteristic ions for DAN-Imp2-RED.

Furazidine (FUR):

Furazidine (FUR):

FUR-Imp1-RED:

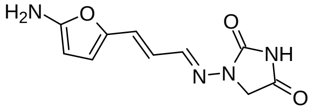

Molecular Formula: $C_{10}H_{10}N_4O_3$
Formula Weight: 234.2114

$C_{10}H_8N_3O_3$; m/z 218:

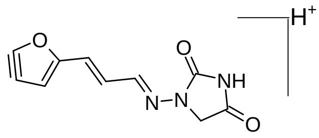

$C_9H_{10}N_3O$; m/z 192:

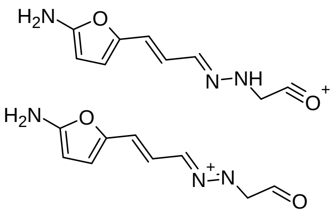

$C_9H_7N_2O_2$; m/z 175:

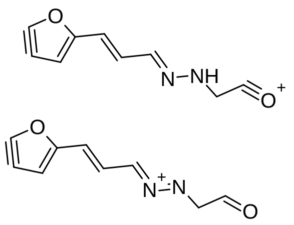

$C_8H_7N_2O$; m/z 147:

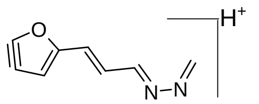

$C_7H_7N_2O$; m/z 135:

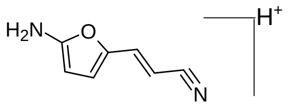

C_7H_5NO; m/z 119:

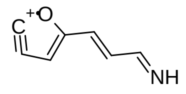

C_6H_6NO; m/z 108:

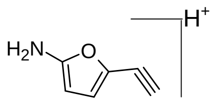

Fig. S32. The proposed molecular structures of characteristic ions for FUR-Imp1-RED.

Furazidine (FUR):

FUR-Imp2-RED:

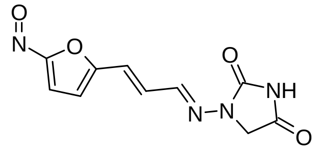

Molecular Formula: $C_{10}H_8N_4O_4$
Formula Weight: 248.19492

$C_{10}H_8N_3O_4$; m/z 234:

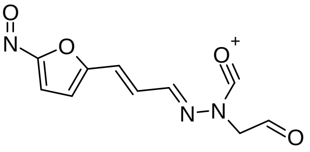

$C_9H_8N_3O_3$; m/z 206:

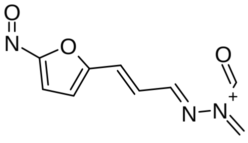

m/z 178:

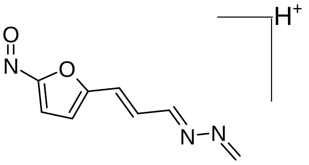

$C_7H_5N_2O_2$; m/z 149:

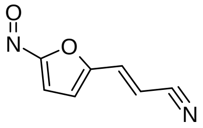

$C_7H_5N_2O$; m/z 133:

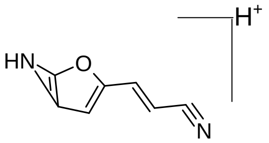

$C_4H_4N_3O_2$; m/z 126:

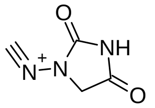

$C_6H_4NO_2$; m/z 122:

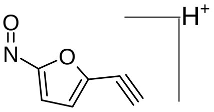

C_7H_6NO; m/z 120:

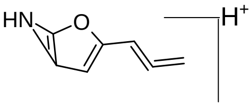

$C_6H_5N_2$; m/z 105:

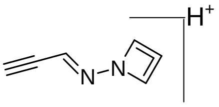

$C_3H_5N_2O_2$; m/z 101:

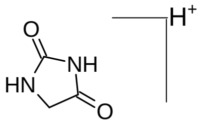

C4H2NO; m/z 80:

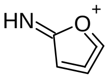

Fig. S33. The proposed molecular structures of characteristic ions for FUR-Imp2-RED.

Furazidine (FUR):

Furazidine (FUR):

FUR-Imp3-RED:

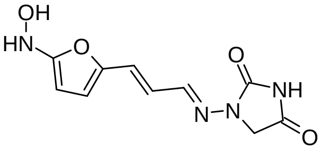

Molecular Formula: $C_{10}H_{10}N_4O_4$
Formula Weight: 250.2108

$C_{10}H_{10}N_4O_3$; m/z 234:

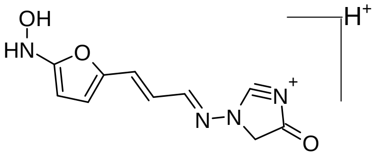

$C_9H_8N_3O_3$; m/z 206:

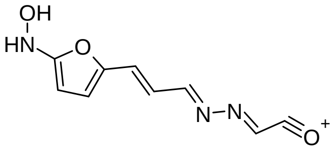

$C_9H_7N_2O_2$; m/z 175:

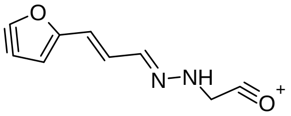

$C_7H_5N_2O_2$; m/z 149:

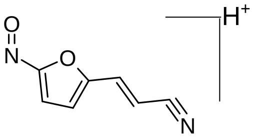

$C_8H_7N_2O$; m/z 147:

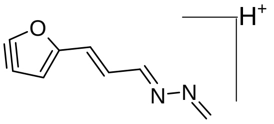

$C_7H_5N_2O$; m/z 133:

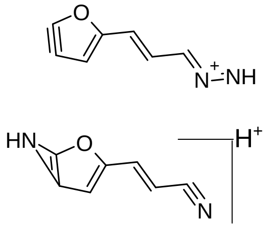

C_7H_6NO; m/z 120:

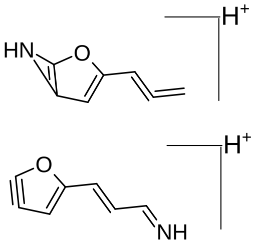

$C_3H_4N_3O_2$; m/z 114:

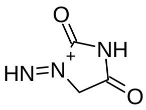

C_6H_6NO; m/z 108:

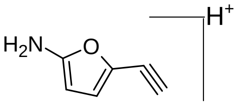

$C_3H_5N_2O_2$; m/z 101:

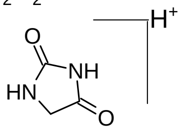

Fig. S34. The proposed molecular structures of characteristic ions for FUR-Imp3-RED.

Nitrofural (NF):

NF-Imp1-RED:

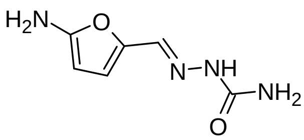

Molecular Formula: $C_6H_8N_4O_2$
Formula Weight: 168.15332

$C_5H_6N_3O_2$; m/z 140:

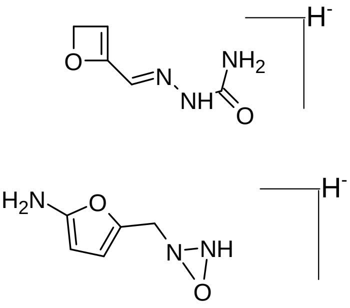

$C_5H_6N_3O$; m/z 124:

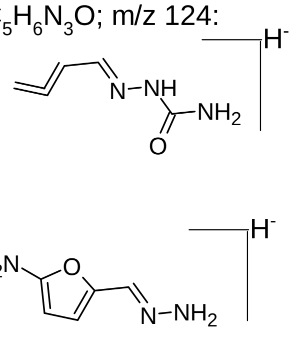

$C_5H_4N_3O$; m/z 122:

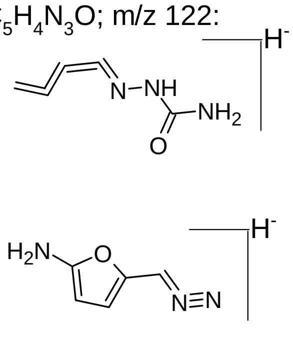

$C_5H_5N_2O$; m/z 109:

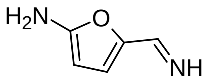

$C_4H_5N_2O$; m/z 97:

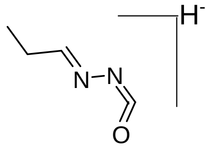

$C_4H_5N_2O$; m/z 95:

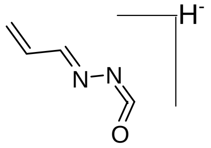

Fig. S35. The proposed molecular structures of characteristic ions for NF-Imp1-RED.

Nitrofural (NF):

Nitrofural (NF):

NF-Imp2-RED:

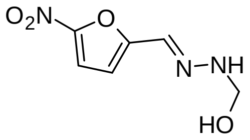

Molecular Formula: $C_6H_7N_3O_4$
Formula Weight: 185.13748

$C_6H_5N_3O_3$; m/z 167:

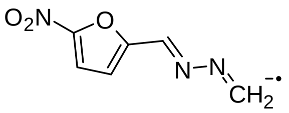

$C_5H_5N_2O_3$; m/z 141:

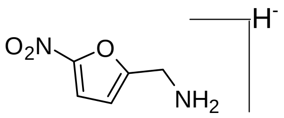

$C_5H_3NO_3$; m/z 125:

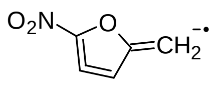

$C_4H_5N_2O$; m/z 97:

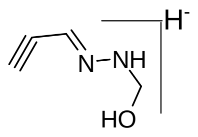

Fig. S36. The proposed molecular structures of characteristic ions for NF-Imp2-RED.

Nitrofurantoin (NFT):

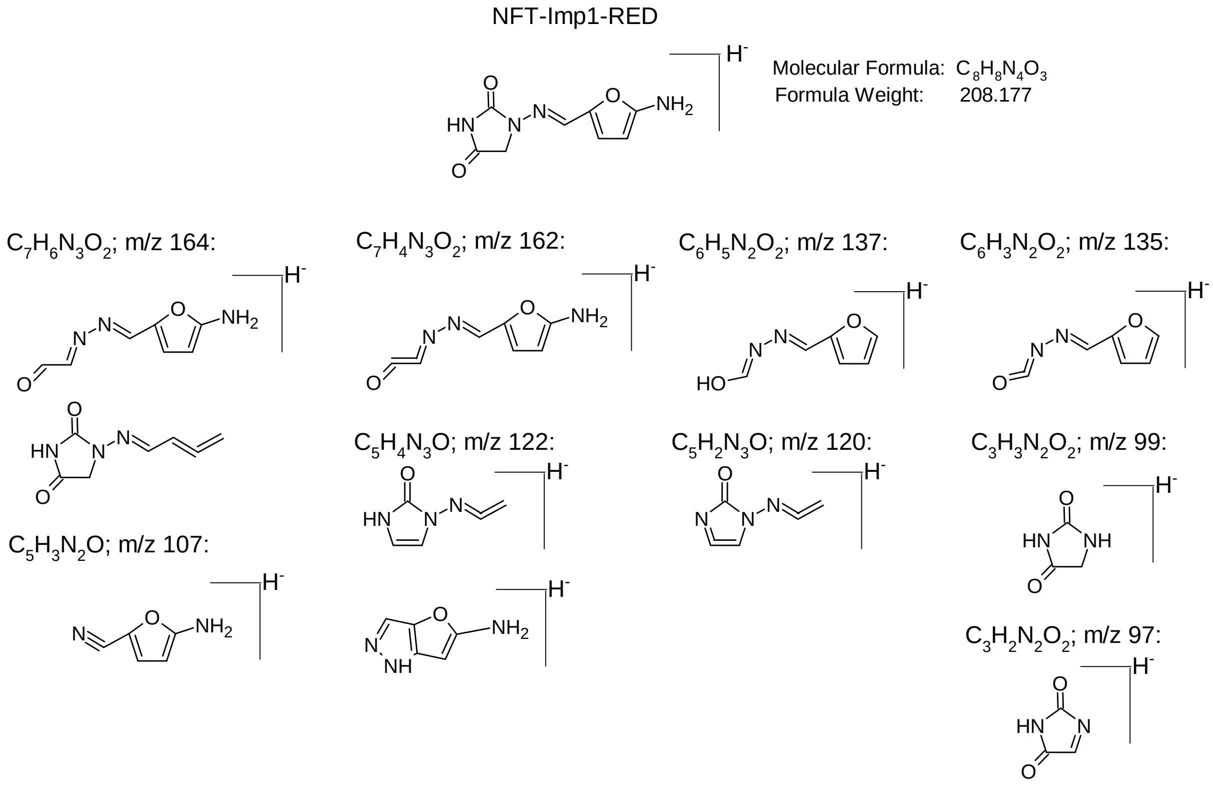

Fig. S37. The proposed molecular structures of characteristic ions for NFT-Imp1-RED.

Nitrofurantoin (NFT):

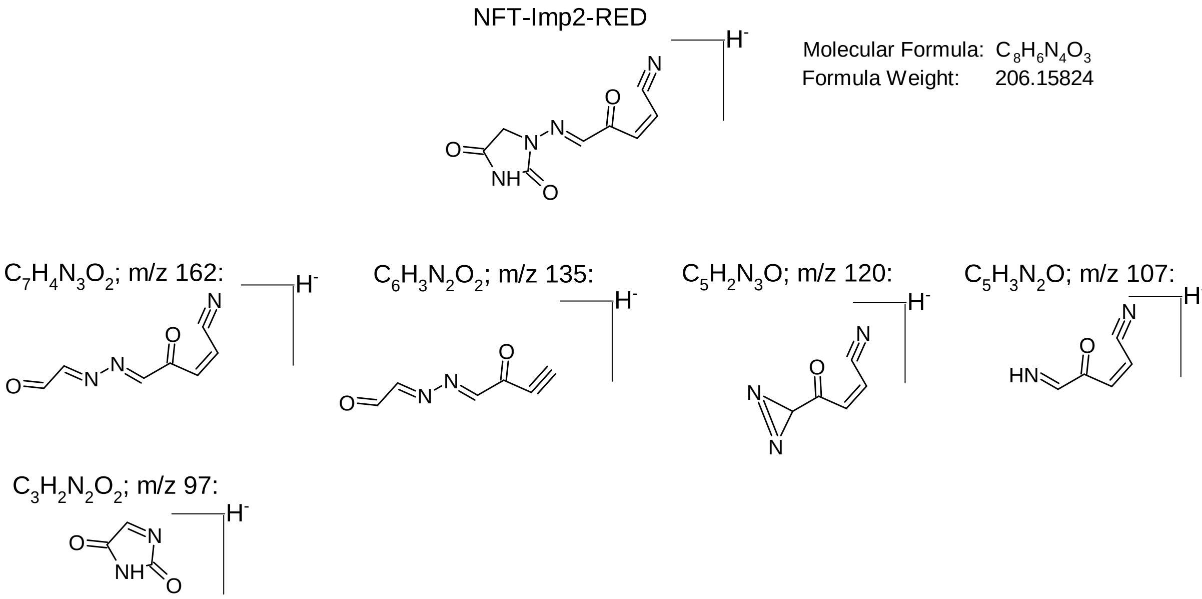

Fig. S38. The proposed molecular structures of characteristic ions for NFT-Imp2-RED.

Nitrofurantoin (NFT):

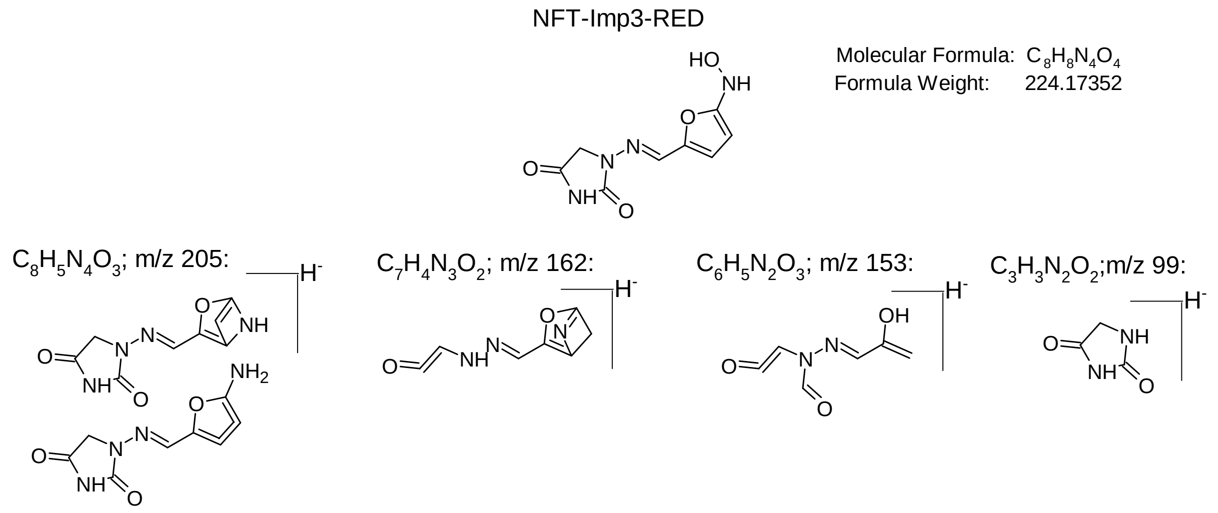

Fig. S39. The proposed molecular structures of characteristic ions for NFT-Imp3-RED.

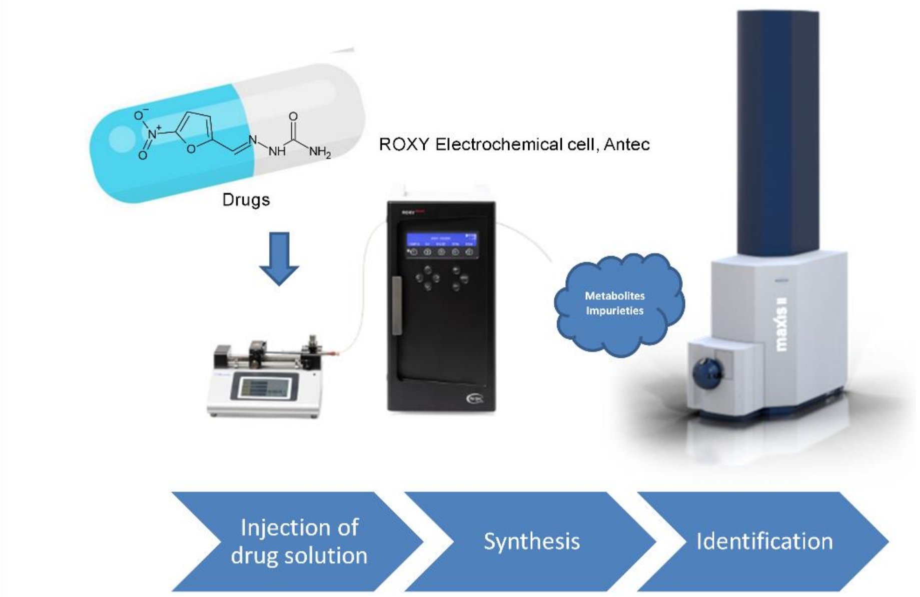

Fig. S40. ROXY™ EC System including a dual syringe infusion pump and the ReactorCell™ connected to electrospray MS.

DFT calculations

The DFT B3LYP/6-311++G(d,p) Gibbs Free Energies, in a.u., were calculated with the optimized geometries and all positive harmonic frequencies with the Gaussian G16 suite of programs and the implemented therein algorithms. The Gibbs Free Energy values are presented in the Tables S1 and S2. They correspond to the Table 1 and Table 2 in the main body. In the stoichiometric equations, the following Gibbs Free Energies, in a.u., of selected smaller molecules were used:

O_2 (singlet) -150.324185

H_2O	-76.454897
H atomic	-0.512911
NH_3	-56.567510

For the reactions using a single oxygen atom we used the (½) O_2 value for the Gibbs Free Energy. For the conversion units we used 1 a.u. = 627.5 kcal/mol.

An example of the red/ox reaction can be written as follows:

$$\text{Reactant} + n\,H^{(+)} + n\,e^{(-)} \rightarrow \text{Product}$$

$$\Delta G\text{ (reaction)} = \Delta G(\text{Product}) - \{\ \Delta G(\text{Reactant}) + n\,\Delta G(\text{H})\ \}$$

where n = 1,2,…, and the H is the hydrogen atom.

Moreover, a compensation of the atomic mass of the left- and the right-hand side of each reaction, i.e., the stoichiometric formulae imposes the same subscripts:

$$C_nH_mN_kO_l \text{ (left-hand side)} = C_aH_bN_cO_d) \text{ (right-hand side)},$$

where $n = a$, $m = b$, $k = c$ and $l = d$.

Table S1. The B3LYP/6-311++G(d,p) calculations. The DAN and FUR – related impurities.

Molecule	Gibbs Free Energy a.u.
DAN	-1134.662612
DAN-Imp1-Ox (A)	-1209.907001
DAN-Imp1-Ox (B)	-1209.899948
DAN-Imp2-Ox	-1285.143066
DAN-Imp1-RED	-1060.640835
DAN-Imp2-RED	-985.460035
FUR	-981.008625
FUR-Imp1-Ox (A)	-1056.245806
FUR-Imp1-Ox (B)	-1056.18473
FUR-Imp1-RED	-831.811081

FUR-Imp2-RED	-905.782939
FUR-Imp3-RED	-906.991357

Table S2. The B3LYP/6-311++G(d,p) calculations. The NF- and NFT – related impurities.

Molecule	Gibbs Free Energy a.u.
NF	-752.145586
NF-Imp1-Ox	-827.393213
NF-Imp1-RED	-602.949676
NF-Imp2-RED	-697.9148
NFT	-903.608193
NFT-Imp1-Ox	-978.845479
NFT-Imp2-Ox	-849.395633
NFT-Imp2-Ox	-1054.091836
NFT-Imp1-RED	-754.413528
NFT-Imp2-RED	-753.206438
NFT-Imp3-RED	-829.593895
